# *AtHKT1* drives adaptation of *Arabidopsis thaliana* to salinity by reducing floral sodium content

**DOI:** 10.1371/journal.pgen.1007086

**Published:** 2017-10-30

**Authors:** Dong An, Jiu-Geng Chen, Yi-Qun Gao, Xiang Li, Zhen-Fei Chao, Zi-Ru Chen, Qian-Qian Li, Mei-Ling Han, Ya-Ling Wang, Yong-Fei Wang, Dai-Yin Chao

**Affiliations:** 1 National Key Laboratory of Plant Molecular Genetics, Chinese Academy of Sciences Center for Excellence in Molecular Plant Sciences, Institute of Plant Physiology and Ecology, Chinese Academy of Sciences, Shanghai, China; 2 College of Agriculture and Biology, Shanghai Jiao Tong University (SJTU), Shanghai, China; 3 University of Chinese Academy of Sciences, Beijing, China; 4 Institute of Plant Stress Biology, State Key Laboratory of Cotton Biology, Department of Biology, Henan University, Kaifeng, China; The Australian National University, AUSTRALIA

## Abstract

*Arabidopsis thaliana high-affinity potassium transporter 1* (*AtHKT1*) limits the root-to-shoot sodium transportation and is believed to be essential for salt tolerance in *A*. *thaliana*. Nevertheless, natural accessions with ‘weak allele’ of *AtHKT1*, e.g. Tsu-1, are mainly distributed in saline areas and are more tolerant to salinity. These findings challenge the role of *AtHKT1* in salt tolerance and call into question the involvement of *AtHKT1* in salinity adaptation in *A*. *thaliana*. Here, we report that *AtHKT1* indeed drives natural variation in the salt tolerance of *A*. *thaliana* and the coastal *AtHKT1*, so-called weak allele, is actually hyper-functional in reducing flowers sodium content upon salt stress. Our data showed that *AtHKT1* positively contributes to saline adaptation in a linear manner. Forward and reverse genetics analysis established that the single *AtHKT1* locus is responsible for the variation in the salinity adaptation between Col-0 and Tsu-1. Reciprocal grafting experiments revealed that shoot *AtHKT1* determines the salt tolerance of Tsu-1, whereas root *AtHKT1* primarily drives the salt tolerance of Col-0. Furthermore, evidence indicated that Tsu-1 *AtHKT1* is highly expressed in stems and is more effective compared to Col-0 *AtHKT1* at limiting sodium flow to the flowers. Such efficient retrieval of sodium to the reproductive organ endows Tsu-1 with stronger fertility compared to Col-0 upon salt stress, thus improving Tsu-1 adaptation to a coastal environment. To conclude, our data not only confirm the role of *AtHKT1* in saline adaptation, but also sheds light on our understanding of the salt tolerance mechanisms in plants.

## Author summary

Identifying the genetic variation driving plant adaptation to salinity is critical for understanding natural selection and evolutionary mechanisms. In this study, we have revealed that the gene *AtHKT1* drives natural variation in the adaptation of *A*. *thaliana* to salinity. Our evidences directly show that the *AtHKT1* allele in the coastal accession Tsu-1 endows *A*. *thaliana* with enhanced adaptability to salinity. Our further experiments have demonstrated that the enhanced adaptability of the coastal accession is attributed to high *AtHKT1* expression in stems, leading to low sodium levels in flowers. Our work not only elucidates the role of *AtHKT1* in local adaptation to salinity but also improves our understanding of salt tolerance mechanisms of plants.

## Introduction

Although plants are sessile and are passively exposed to varying environments, they can successfully complete their life cycle and disperse across a range of environmental conditions. These capabilities are attributable to their physiological plastic and complex gene regulation networks, as well as to their affluent genetic diversities. Therefore, revealing the genetic variation that drives adaptation to particular environments is critical for understanding natural selection and evolutionary mechanisms. Numerous studies have elucidated the genetic changes in different plant species in their adaptation to extreme temperature [1], flowering time [2], photoperiod [3], and microbial attack [4]. Soil is a key environmental factor in maintaining plant growth and a major driving force in plant evolution. Nevertheless, little progress has been made in determining the genetic basis underlying plant adaptation to different edaphic conditions.

Sodium is a non-essential element that is toxic to most plants. Saline soil is an important driver of genetic variation. A previous study has shown that genome duplication alters fitness to allow plants to adapt to saline environments [5]. Nonetheless, only very limited evidence supports the involvement of a specific gene in local adaptation to saline habitats.

*HKT1* (*High-affinity Potassium Transporter*) was first isolated from wheat and was shown to function as a K^+^/Na^+^ symporter [6]. Its homologs in various species, such as *AtHKT1*, *OsHKT1;1* and *SKC1*/*OsHKT1;5*, are generally expressed in xylems of different organs and contribute to plant salt tolerance by unloading sodium from the transpiration stream [7–11]. Interestingly, natural variation in these *HKT* homologs has been observed in both *A*. *thaliana* and rice populations. Four amino acid substitutions in *SKC1/OsHKT1;5* significantly increase its sodium transport activity, restricting sodium flux to leaves and greatly enhancing the salt tolerance of the salt-tolerant rice variety Nona Bokra [9].

*A*. *thaliana* is widely distributed in all kinds of geographical environments, and many ecotypes have adapted to saline conditions [12]. Tsu-1 and Ts-1 are two coastal *A*. *thaliana* accessions that are highly tolerant to salt stress [13]. However, unlike the salt-tolerant rice variety Nona Bokra, they accumulate very high sodium in their leaves. Genetic analysis has revealed that the high leaf sodium phenotypes of Tsu-1 and Ts-1 are controlled by the *AtHKT1* locus [13]. *AtHKT1* is expressed at extremely low levels in the roots of these two accessions due to the absence of a distal enhancer [14]. Genome-wide association studies (GWAS) have confirmed that *AtHKT1* controls the natural variation of leaf sodium content in *A*. *thaliana* populations worldwide and have revealed that *A*. *thaliana* accessions with weak *AtHKT1* alleles are predominantly distributed in coastal or saline areas [15]. These results, together with other molecular ecology evidence, suggest that *AtHKT1* plays a negative role in the salinity adaptation of *A*. *thaliana* [12]. However, this conclusion seemingly conflicts with the observation that the null *hkt1-1* mutant is hypersensitive to salt stress [16–18]. Solving this puzzle is particularly important because it may uncover the role of *AtHKT1* in local adaptation, improve our knowledge of the salt tolerance mechanisms of plants and help resolve the long debate about the relationship between shoot sodium content and salt tolerance.

Edaphic salinity threatens plants via osmotic stress and ion toxicity. Halophyte plants generally accumulate sodium in their shoots, as high sodium helps cells resist osmotic stress, and the sodium is not toxic to the cell as long as it is sequestered in the vacuole [19]. Although *A*. *thaliana* is not a halophyte, the low expression of *AtHKT1* and the high leaf sodium phenotype of coastal salt-tolerant accessions might suggest that *A*. *thaliana* may use a strategy similar to halophytes to adapt to saline habitats. It was therefore hypothesized that *AtHKT1* might serve as a double-edged sword for salt tolerance, while weak expression of *AtHKT1* could well balance the osmotic and ionic toxicity of high salt for the best salt tolerance [13,15]. Alternatively, *AtHKT1* might contribute to saline adaptation in an uncharacterized mechanism. Otherwise, it should be independent of the saline adaptation.

Seed production is a key index used for evaluating crops economic efficiency in agriculture and is an essential indicator in assessing the evolutionary adaptation of plants [20]. Therefore, in this study, we used seed yield as a central index for estimating the adaptability of plants. We found that *AtHKT1* positively drives plant adaptation to a coastal environment, by changing its expression pattern to efficiently retrieve sodium from the stem-to-flower xylem sap and thus reduce floral sodium content to increase fecundity upon salt stress.

## Results

### Salt tolerance of *A*. *thaliana* has a linear positive relationship with expression levels of *AtHKT1*

**Fig 1:**
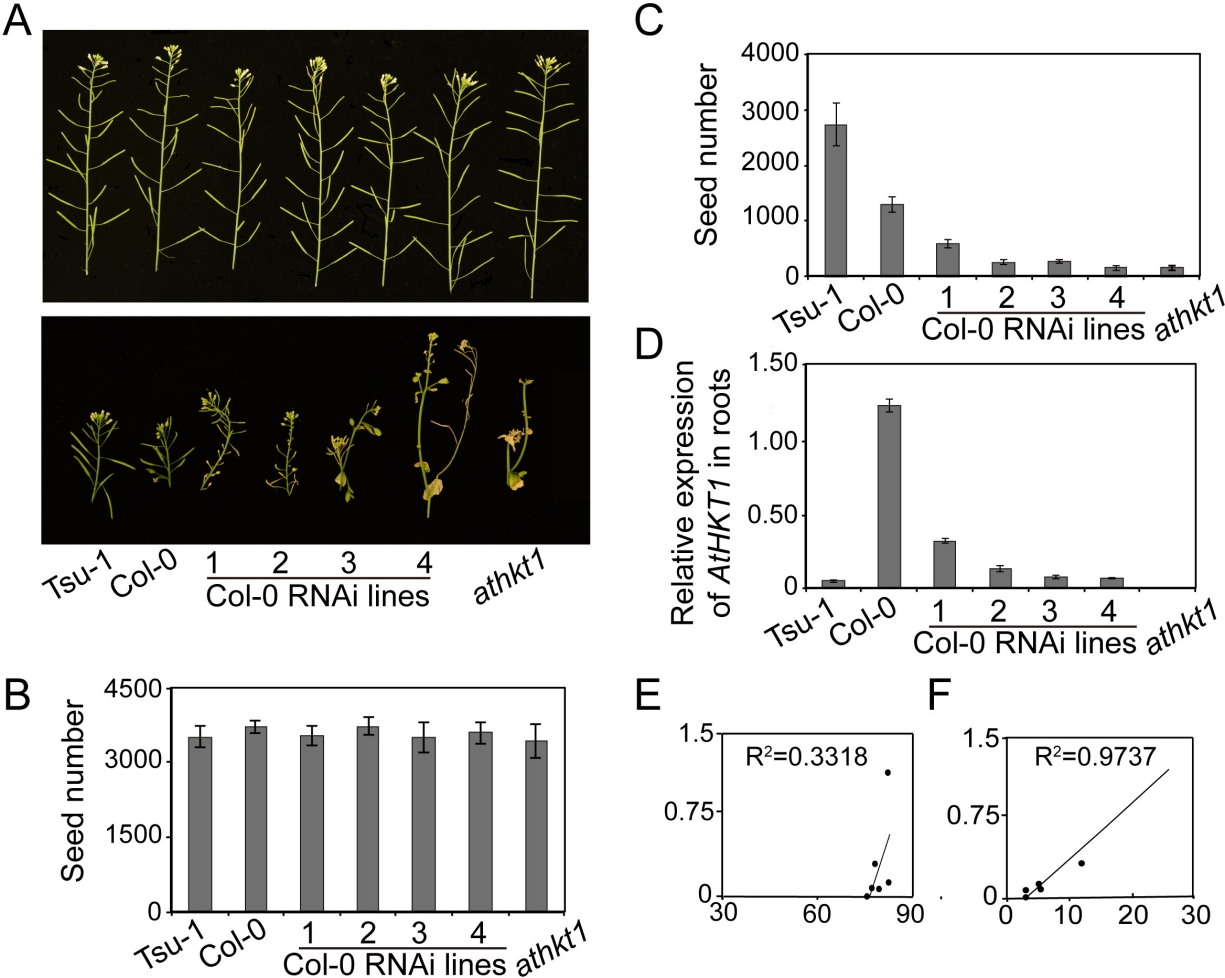
*AtHKT1* contributes to salt tolerance in a linear manner. (A) Flowers and siliques of Tsu-1, Col-0, Col-0 *AtHKT1* RNAi lines, and *athkt1* under normal conditions (upper) or treated with 100 mM NaCl (lower) for two weeks. (B-C) Numbers of seeds from the plants shown in (A) under normal (B) and 100 mM NaCl treatment conditions (C). Two-week-old plants were treated without or with twice 100 mM NaCl, and seeds were collected continuously during silique maturation. (D) *AtHKT1* expression level assessed by qRT-PCR in the roots of 4-week-old Tsu-1, Col-0, Col-0 *AtHKT1* RNAi lines, and *athkt1* grown in liquid culture. (E-F) Correlation of seed number and *AtHKT1* expression level under normal (E) and salt stress conditions (F). Data represented with mean ± SE, *n* = 12, significant differences were determined by ANOVA.

To examine if there is an expression balance of *AtHKT1* for the best salt tolerance, independent knockdown lines of *AtHKT1* in the Col-0 background were established. We also isolated an *athkt1* null mutant in the Col-0 background as a positive control by backcrossing *hkt1-1* with Col-0. Under normal condition, no difference on fecundity was observed among different genotypes (Fig 1A and 1B). Though the coastal saline-adapted accession Tsu-1 produced twice as many seeds as Col-0, whereas the *athkt1* knockout mutant yielded almost no seeds when they were treated with 100 mM NaCl, a mild salt stress condition that mimics the natural saline habitat of coastal *A*. *thaliana* accessions (Fig 1A and 1C). These data indicate that *AtHKT1* is essential for salt tolerance and that Tsu-1 is more adapted to saline soil. Moreover, the number of seeds in *AtHKT1* knockdown lines declined with decreases in *AtHKT1* levels, while those expressing *AtHKT1* to a similar level as Tsu-1 produced a similar number of seeds as *athkt1* (Fig 1A, 1C and 1D). In addition, we found that the seed number is positively and linearly correlated with *AtHKT1* expression when exposed to salt stress (Fig 1F), but not under normal condition (Fig 1E). To sum up, these data demonstrate that *AtHKT1* linearly contributes to salt tolerance, and that weak expression of *AtHKT1* does not really balance the osmotic and ionic toxicities for the best salt tolerance.

### *AtHKT1* is responsible for the saline adaptation phenotype of Tsu-1

A rough mapping has shown that the salt tolerance phenotype of Tsu-1 is linked to the *AtHKT1* locus [13]. Nonetheless, it remained unclear whether *AtHKT1* itself, or a locus linked to *AtHKT1*, controls the phenotype. To address this question, we tried to map the responsible locus or loci by QTL analysis. Epistatic interaction and additive effects generally cause a big problem for mapping QTLs. Fixing genotype of one causal locus could remove or minimize these problems, therefore we synthesized an F2 population derived from a cross between Tsu-1 and *athkt1*, as both alleles are hypo-functional.

**Fig 2:**
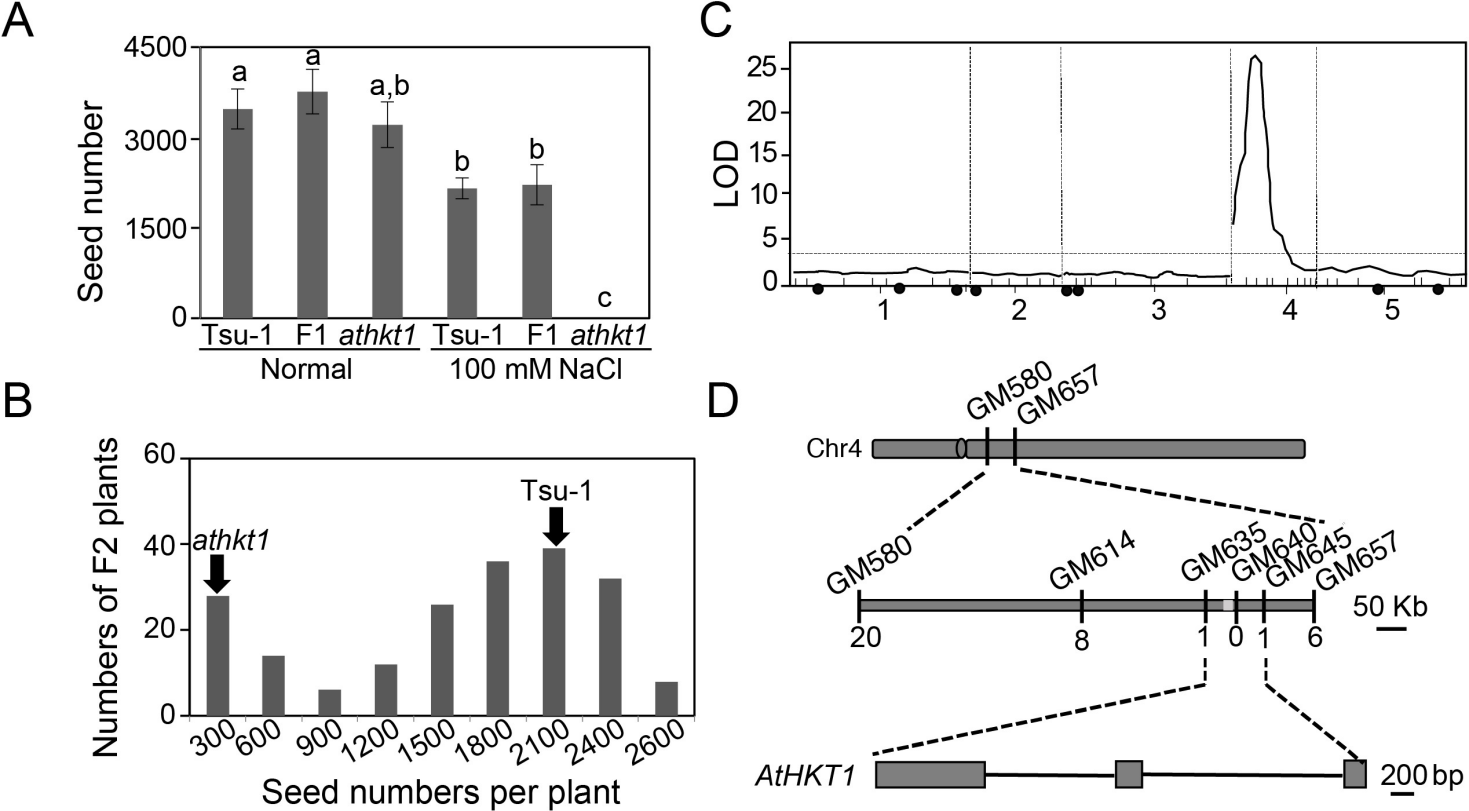
QTL analysis of salt tolerance loci in Tsu-1 mapped to a single major region where *AtHKT1* is located. (A) Numbers of seeds of F1 plants derived from a cross between Tsu-1 and *athkt1* under normal and salt stress conditions. Data represented with mean ± SE, *n* = 12, and significant differences were determined by ANOVA. (B) Numbers of seeds of 201 F2 plants subjected to salt treatment. (C) QTL analysis of the F2 population using the R/qtl package. The threshold of LOD indicated by the dotted line was 2.5. The black dots indicate the genetic locations of 8 NHX genes. (D) Fine mapping of 537 F2 plants using 10 newly developed markers narrowed down the location of the causal gene to a region between GM635 and GM645 on chromosome 4 that contains *AtHKT1*. The number underlined in the middle line indicates recombination events.

We first examined the productivity of the F1 progeny and observed that F1 plants produced an amount of seeds similar to Tsu-1 when exposed to 100 mM NaCl (Fig 2A), suggesting that the adaptive trait is probably controlled by one or more dominant loci. An analysis of 201 F2 individuals further confirmed that a single major locus controls saline adaptation, given that the ratio of salt tolerant F2s (153 individuals) and salt sensitive F2s (48 individuals) conforms to 3:1 (Χ^2^ = 0.08<Χ^2^_0.05_ = 3.84) (Fig 2B). To identify the locus, we performed a QTL analysis using 36 polymorphic genetic markers covering all five chromosomes. As expected, we identified a single major QTL on chromosome 4 that contributed to 53.1% of the variance in seed yield under salt stress (Fig 2C). Interestingly, this candidate region contains *AtHKT1*, but none of the NHX genes that could sequester sodium in the vacuole [21].

We employed an enlarged F2 population with 537 individuals and developed 10 polymorphic markers in the candidate region for fine mapping. Based on genotype and phenotype analyses of the F2 individuals or their F3 progenies, we narrowed down the causal gene to a 100-kb region between genetic markers GM635 and GM645 (Fig 2D). This region contains only 26 annotated genes; and only *AtHKT1* is related to salt tolerance (S1 Table), suggesting that the *AtHKT1* is the causal locus for adaptation of Tsu-1 to saline conditions. To verify this, we knocked out *AtHKT1* in Tsu-1 using CRISPR (clustered regulatory interspaced short palindromic repeats)-Cas9 (CRISPR-associated protein 9) technology [22]. In the T2 generation, we obtained two homologous knockout mutants of *AtHKT1* and named them *hkt1-c1* and *hkt1-c2* (*c* stands for alleles generated by CRISPR-Cas9 technology). In *hkt1-c1*, *AtHKT1* has a thymine inserted between the 450^th^ and 451^st^ nucleotides of the coding sequence (S1A Fig), while, in the *hkt1-c2*, four nucleotides (from the 447^th^ to the 450^th^ nucleotides after the start codon) are deleted (S1B Fig), both of which result in frame shifts.

**Fig 3:**
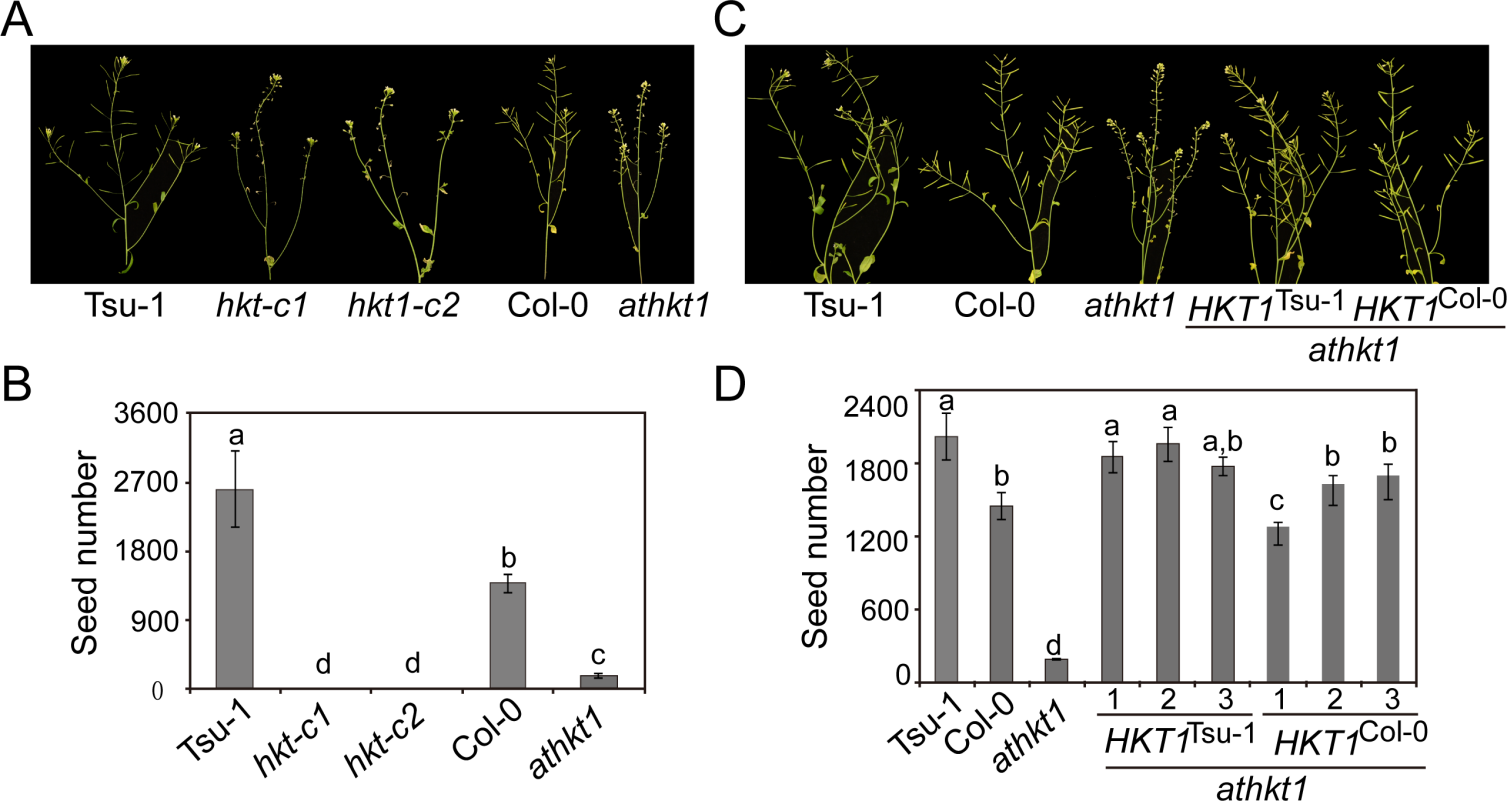
A single *AtHKT1* locus is sufficient to drive natural variation in salinity adaptation. (A) Flowers and siliques of Tsu-1, *hkt1-c1*, *hkt1-c2*, Col-0, and *athkt1* subjected to salt treatment. (B) Numbers of seeds of the plants shown in (*A*) under salt treatment (*n* = 12). (C) Flowers and siliques of Tsu-1, Col-0, *athkt1*, and *athkt1* complementation lines with *AtHKT1* fragment from Tsu-1 or Col-0 following salt treatment. (D) Number of seeds of the plants shown in (C) under salt treatment. (*n* > 40). The plants were treated with 100 mM NaCl twice after growth for 2 weeks. Data represented with mean ± SE; significant differences were determined by ANOVA.

Under normal conditions, there were no differences on seed production among *hkt1-c1*, *hkt1-c2* and Tsu-1 (S1C Fig). Yet, when treated with 100 mM NaCl, the two mutants were totally sterile, whereas the wild control Tsu-1 was still productive (S1D Fig). The phenotypes of the two mutants were comparable to or even more severe than that of *athkt1*, as *athkt1* still produced some seeds, although considerably less than Col-0 under the same conditions (Fig 3A and 3B). Considering that Tsu-1 produced significantly more seeds than Col-0 in the same salt treatment experiment (Fig 3A and 3B), we conjectured that *AtHKT1* is responsible for the natural variation in saline adaptation between Tsu-1 and Col-0. To further confirm that the phenotypes of *hkt1-c1* and *hkt1-c2* were caused by *AtHKT1* knock-out, we crossed them with *athkt1* respectively. The F1 plants were all sterile when treated with 100 mM NaCl (S2A and S2B Fig), verifying that the phenotypes of *hkt1-c1* and *hkt1-c2* were not an off-target effect.

To further determine whether a single *AtHKT1* locus is sufficient to drive the variation in the salinity adaptation of *A*. *thaliana*, we cloned the genomic DNA of *AtHKT1*, including its long promoter from Col-0 and Tsu-1, and introduced each construct into the null *athkt1* mutant separately. We tested the salt tolerance of three independent transgenic lines for each construct by assessing productivity. Transgenic lines with either the Col-0 *AtHKT1* fragment or the Tsu-1 fragment could complement the salt-hypersensitive phenotype of *athkt1* (Fig 3C). Nevertheless, the Col-0 *AtHKT1* fragment could only restore the saline adaptability of *athkt1* to the Col-0 level, whereas the Tsu-1 fragment was sufficient to restore *athkt1* to the Tsu-1 level of adaptability (Fig 3D). These data demonstrated that a single *AtHKT1* locus is sufficient to drive the natural variation in the salinity adaptation of Col-0 and Tsu-1.

### Reciprocal grafting reveals that the salt tolerance phenotype of Tsu-1 is driven by shoot *AtHKT1*, whereas that of Col-0 is driven by root *AtHKT1*

**Fig 4:**
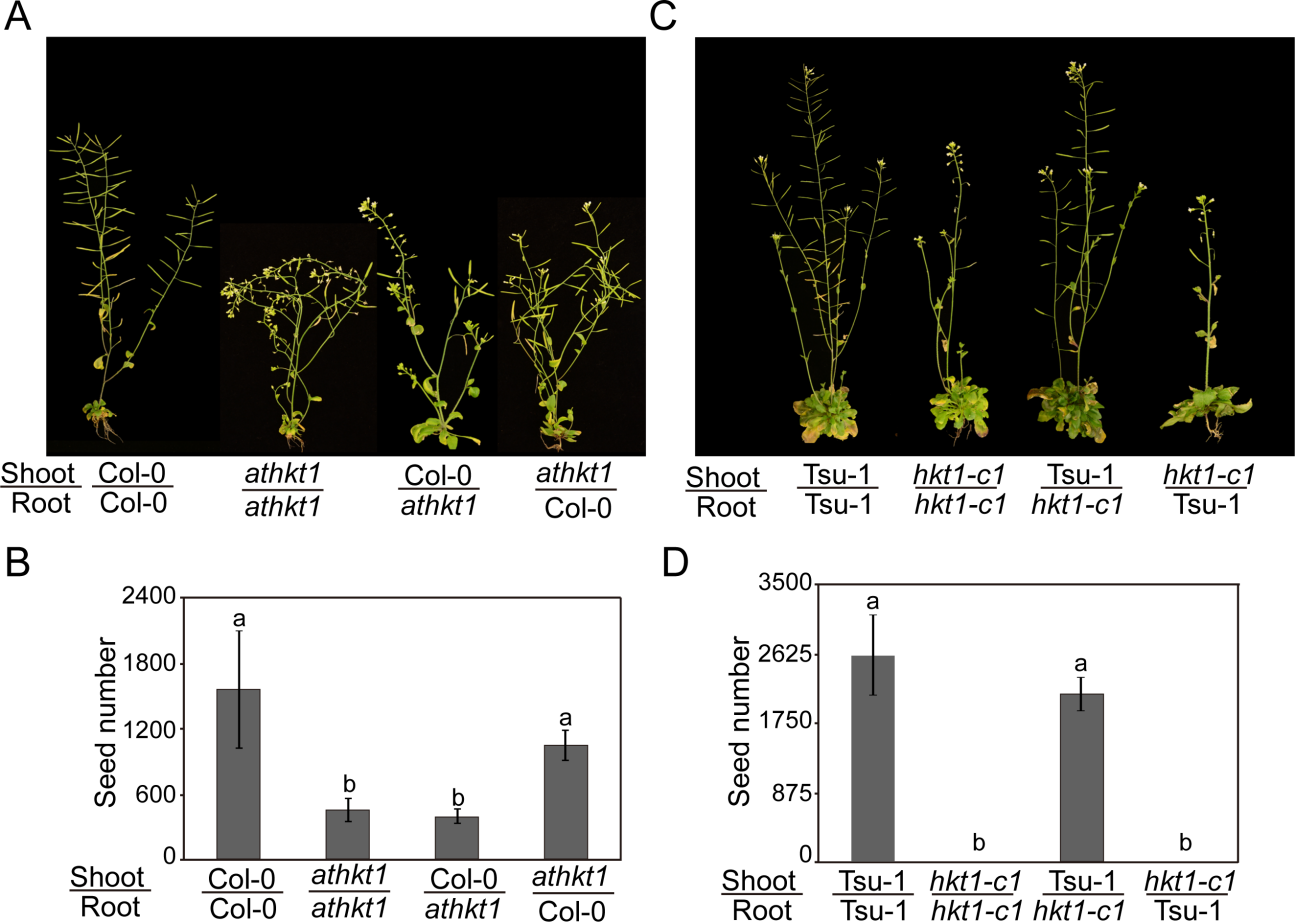
Reciprocal grafting experiments revealed that the salt tolerance phenotype of Tsu-1 is driven by shoot *AtHKT1*, whereas the phenotype of Col-0 is driven by root *AtHKT1*. (A) Plants generated via reciprocal graft between Col-0 and *athkt1* treated with 100 mM NaCl for 2 weeks. (B) Numbers of seeds from reciprocally grafted Col-0 and *athkt1* plants treated with 100 mM NaCl. (C) Reciprocally grafted plants between Tsu-1 and *hkt1-c1*subjected to 100 mM NaCl for 2 weeks. (D) Numbers of seeds from reciprocally grafted Tsu-1 and *hkt1-c1* plants subjected to 100 mM NaCl. Successfully grafted plants (*n* > 12) were transferred to pots for 2 weeks following 100 mM NaCl treatment twice for 2 weeks before images were taken. Data represented with mean ± SE; significant differences were determined by ANOVA.

Root *AtHKT1* expression drives leaf sodium content in both Col-0 and Tsu-1 [13]. Nevertheless, it was unclear in what tissue *AtHKT1* drives salt tolerance in these ecotypes. To address this question, we performed two reciprocal grafting experiments; one between *athkt1* and Col-0, and the other was between *hkt1-c1* and Tsu-1. We observed that the grafted plants with *athkt1* as the shoot, and Col-0 as the root, were as tolerant as self-grafted Col-0 plants; whereas the grafted plants with Col-0 as the shoot, and *athkt1* as the root, were similar to self-grafted *athkt1* plants (Fig 4A and 4B). This result indicates that salt tolerance of Col-0 predominantly attributes to root *AtHKT1*. However, the grafting experiment between Tsu-1 and *hkt1-c1* showed the opposite results. The grafted plants with Tsu-1 scion and *hkt1-c1* stock produced a similar salt tolerant phenotype as Tsu-1 self-grafted plants, whereas *hkt1-c1* self-grafted plants and grafted plants with *hkt1-c1* scion and Tsu-1 stock were sterile upon salt stress (Fig 4C and 4D). These data showed that the shoot expression of *AtHKT1* drives the saline adaptation of Tsu-1. Moreover, they suggest that although Tsu-1 *AtHKT1* is hypo-functional in roots, it is hyper-functional in shoots.

### Tsu-1 *AtHKT1* is hyper-functional in stems due to variations in expression

**Fig 5:**
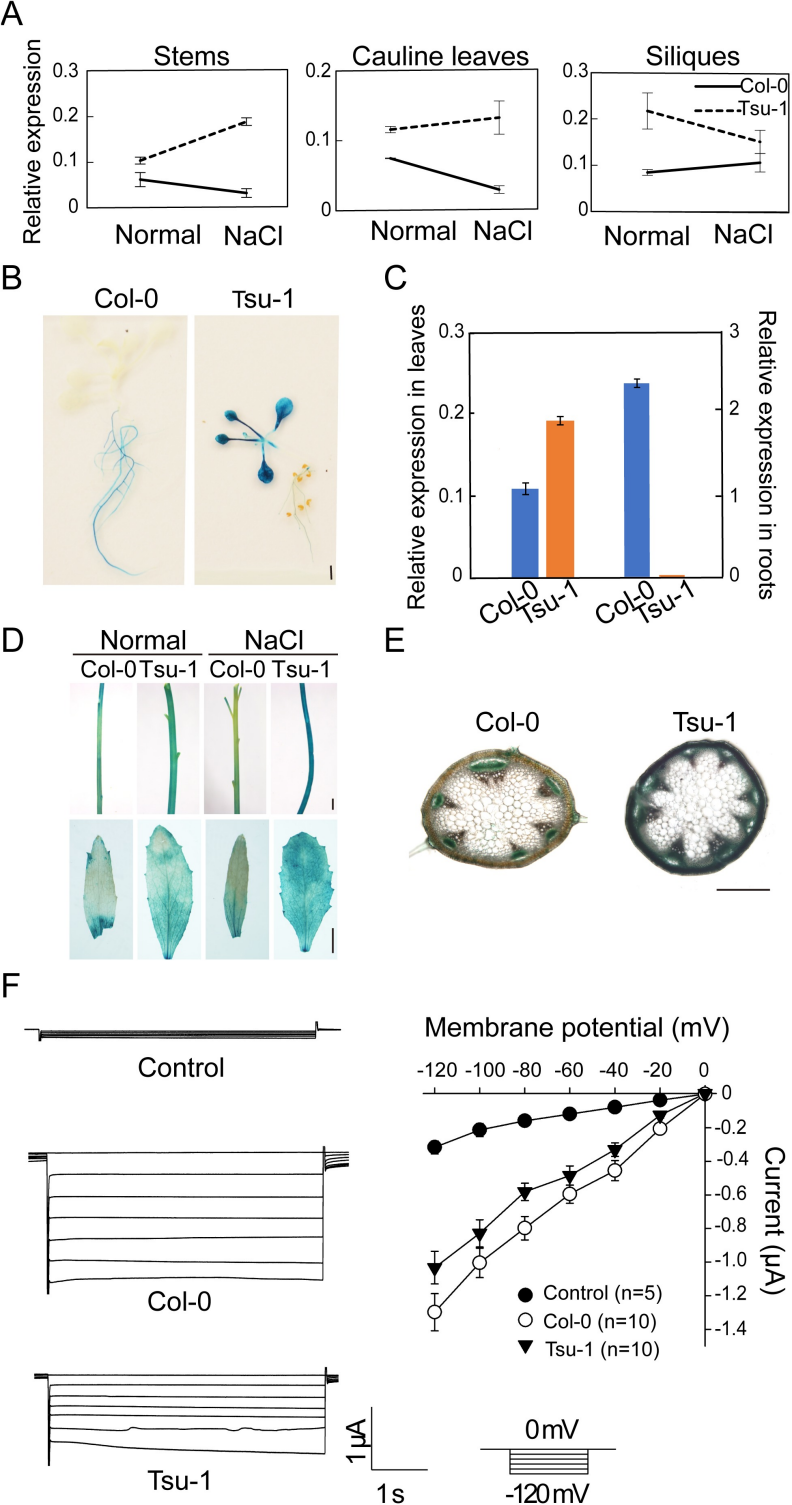
*AtHKT1* is hyper-functional in Tsu-1 stems. (A) *AtHKT1* expression in the stems, cauline leaves, and siliques of 4-week-old Tsu-1 and Col-0 before and after 100 mM NaCl treated for 24 h. Data represented with mean ± SE, *n* = 6. (B) GUS staining of 2-week-old T1 transgenic seedlings expressing *pAtHKT1*:*GUS* from Col-0 and Tsu-1. (Scale bar: 0.5 cm). (C) *AtHKT1* expression in the shoots and roots of Tsu-1 and Col-0. Two-week-old seedlings grown on 1/2 MS medium. (D) GUS staining of the stems and cauline leaves of transgenic lines expressing *pAtHKT1*:*GUS* from Col-0 and Tsu-1. Four-week-old transgenic plants grown in pots before (Normal) and after 100 mM NaCl treatment for 24 h (NaCl). (Scale bars: 0.5 cm). (E) GUS staining of the stems of 4-week-old transgenic lines expressing *pAtHKT1*:*GUS* from Col-0 (upper) and Tsu-1 (lower) by hand sectioning. (Scale bar: 250 μm). (F*)* Electrophysiological recording of the activity of AtHKT1 from Tsu-1 and Col-0. The current-voltage curves were measured using a ramp command in which the membrane potential was stepped from -120 mV to 0 mV. The data shown here are the steady-state current-voltage curve (mean ± SE) in the bath solution containing 10 mM Na^+^ and 0.3 mM K^+^.

The grafting results inspired us to analyze the expression of *AtHKT1* in different aerial tissues. Quantitative reverse transcription-polymerase chain reaction (qRT-PCR) showed that differences in *AtHKT1* expression between Tsu-1 and Col-0 depend on the tissue and the treatment. *AtHKT1* was expressed at similar levels in the rosette leaves of 4-week-old of Tsu-1 and Col-0 when exposed to 100 mM NaCl for 24 h, but its expression was higher in the rosette leaves of Col-0 than of Tsu-1 under normal conditions (S3A Fig). In flowers, Tsu-1 expressed *AtHKT1* at a similar level as Col-0 under normal conditions but it expressed less than Col-0 when treated with salt (S3B Fig). Interestingly, Tsu-1 expressed significantly higher *AtHKT1* in the stems, cauline leaves and siliques compared to Col-0 (Fig 5A). When treated with salt, Tsu-1 expressed 6 times more *AtHKT1* than did Col-0 in the stems, which is consistent with the hypothesis that Tsu-1 allele of *AtHKT1* is hyper-functional in shoot.

To confirm the qRT-PCR results and further examine the spatial expression patterns of *AtHKT1* in detail, we generated transgenic Col-0 plants carrying a β-glucuronidase (GUS) reporter driven by the long promoter of *AtHKT1* from either Tsu-1 or Col-0. We examined 5 independent transgenic lines expressing *pAtHKT1*^*Tsu-1*^::*GUS* and 7 independent lines expressing *pAtHKT1*^*Col-0*^::*GUS*. The transgenic lines containing the same construct showed consistent GUS signals. At the seedling stage, all 5 lines expressing *pAtHKT1*^*Tsu-1*^::*GUS* exhibited very weak GUS activity in the roots, whereas 6 of the 7 lines expressing *pAtHKT1*^*Col-0*^::*GUS* strongly expressed GUS in the stele of the roots (Fig 5B). These observations were consistent with previous findings [14] and with our qRT-PCR results (Fig 5C), indicating that the constructs were correctly expressed in Col-0 plants. Contrary to the root results, the GUS signal in stems and cauline leaves of 4-week-old of plants was much stronger in Tsu-1 than in Col-0 (Fig 5D). This result further confirmed the qRT-PCR results.

We further examined the detailed expression site(s) of *AtHKT1* in stems by hand sectioning of the *pAtHKT1*:*GUS* transgenic lines. The GUS signal driven by the Tsu-1 *AtHKT1* promoter was mainly observed in the xylem of the stem (Fig 5E), indicating that Tsu-1 *AtHKT1* predominantly functions in removing sodium from the flux to the flowers. By contrast, the GUS signal driven by Col-0 *AtHKT1* promoter was very weak in the xylem and was observable in the phloem (Fig 5E). These data demonstrated that variation in the *AtHKT1* promoter affects not only the expression level but also the spatial specificity of *AtHKT1* expression.

In addition to large insertion/deletion (indel) polymorphisms in the promoter region (S4A Fig), multiple single nucleotide polymorphisms (SNPs) were also observed in the coding sequence with several affecting the amino acid sequence (S4B Fig). To test whether the amino acid changes in AtHKT1 in Tsu-1 affect its transport activity, we expressed AtHKT1 protein from Tsu-1 and Col-0 in *Xenopus laevis* oocytes for electrophysiological analysis. As expected, we detected sodium transporter activity of AtHKT1 (Fig 5F), but there was no significant difference between the proteins from Col-0 and Tsu-1, indicating that the amino acid changes do not affect AtHKT1 activity. This observation is consistent with previous findings that these changed amino acids are not in conserved regions of AtHKT1 [23,24].

### Tsu-1 *AtHKT1* is more effective in limiting sodium accumulation in flowers exposed to salt stress

**Fig 6:**
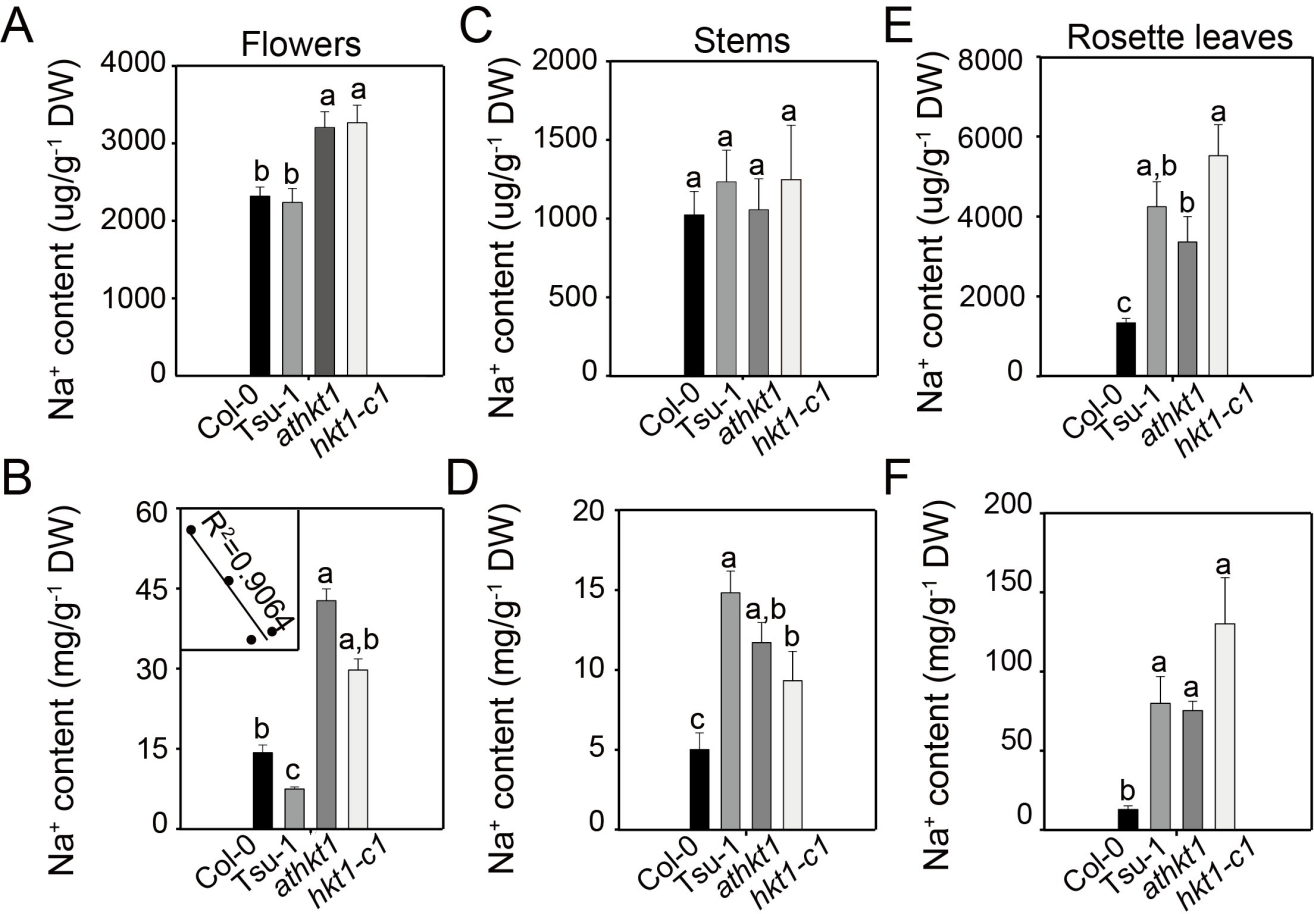
Na^+^ content of Col-0, Tsu-1, *athkt1* and *hkt1-c1*. (A) Na^+^ content in flowers under normal conditions. (B) Na^+^ content in flowers under salt treatment. Negatively correlated between floral sodium content and seed number under salt stress (*Embedded*) (C) Na^+^ content in stems under normal conditions. (D) Na^+^ content in stems under salt treatment. (E) Na^+^ content in rosette leaves under normal conditions. (F) Na^+^ content in rosette leaves under salt treatment. Five-week-old plants irrigated without or twice with 100 mM NaCl were used to analyze Na^+^ by ICP-MS. Data represented with mean ± SE, *n* = 12, and significant differences were determined by ANOVA.

The increased expression of *AtHKT1* in the xylem of Tsu-1 stems suggested that Tsu-1 is more effective than Col-0 at limiting sodium flow to flower. To confirm this hypothesis, we measured the sodium content in the flowers. Under normal conditions, *athkt1* accumulated 38% higher sodium in flowers than Col-0, and *hkt1-c1* accumulated 46% higher than Tsu-1, but there were no significant differences between the wild-types and between the mutants (Fig 6A). Nevertheless, the sodium content in Tsu-1 flowers was only 62% of that in Col-0 flowers when exposed to salt treatment (Fig 6B). By contrast, upon salt stress, the loss of *AtHKT1* function in Col-0 resulted in a 199% increase in floral sodium, whereas the loss of *AtHKT1* function in Tsu-1 resulted in a 275% increase in floral sodium (Fig 6B). These data indicated that *AtHKT1* in Tsu-1 stems is more effective in limitation of sodium flux to flowers than *AtHKT1* in Col-0 roots. In addition, we analyzed the relationship between floral sodium content and seed number under salt stress and found these two variables to be negatively correlated (Fig 6B *Embedded*), demonstrating that the sodium content in flowers under salt stress is a determinant of saline adaptation.

An examination of stem sodium content provided additional evidence supporting the idea that Tsu-1 *AtHKT1* is involved in sodium removal in stems. There was no significant difference in stem sodium content among Col-0, Tsu-1, *athkt1* and *hkt1-c1* under normal conditions (Fig 6C). However, under salt stress, Tsu-1 accumulated 59% more sodium in stems than did *hkt1-c1*, whereas Col-0 accumulated 57% less stem sodium than *athkt1* (Fig 6D); these results show that *AtHKT1* in Tsu-1 promotes sodium accumulation in stems and that *AtHKT1* in Col-0 has the opposite effect. This observation further confirmed that Tsu-1 *AtHKT1* is hyper-functional in restricting sodium flow from stems to flowers and that Col-0 *AtHKT1* functions to limit sodium flow from roots to shoots, and explains why the Tsu-1 allele of *AtHKT1* endows the coastal accessions with higher adaptation ability to salinity environment than the inland accessions.

Previous studies have illustrated that the leaf sodium content cross different *A*. *thaliana* accessions under normal conditions is not associated with its salinity tolerance [15]. However, the leaf sodium content under salt stress is unknown. Therefore, we also measured the leaf sodium content under normal conditions and after salt treatment. Consistent with previous studies, Tsu-1 and the two null mutants accumulated much more leaf sodium than Col-0 under normal conditions (Fig 6E). The result was similar when the plants were treated with salt, as the leaf sodium content in Tsu-1 and the two null mutants was much higher than that in Col-0 (Fig 6F).

## Discussion

The salinization of irrigated land is increasingly detrimental to plant reproduction and agricultural productivity, as most plant species are sensitive to high concentrations of sodium. Understanding the genetic and molecular mechanisms underlying salt tolerance is necessary for resolving such problems. Natural variation is an important genetic resource for studying gene function, as well as evolutionary biology. Previous studies have shown that HKT family transporters are essential for natural variation in salt tolerance of rice [9,25,26]. *AtHKT1* has also been found to control natural variation in leaf sodium content of *A*. *thaliana*, but it is still unclear if and how this locus contributes to saline adaptation [13,15]. In this study, we performed a series of experiments to address this question.

### *AtHKT1* positively contributes to saline adaptation of *A*. *thaliana*

As seed number is an essential trait for population expansion [20], we used this trait for assessing saline adaptation of plants. Previous studies have suggested that *AtHKT1* might be fine-tuned for balancing the ionomic and osmotic stresses caused by high salt, as weak alleles of *AtHKT1* might improve salt tolerance, whereas null alleles of *AtHKT1* suppress salt tolerance [13]. Our observation of the linear relationship between salt tolerance and *AtHKT1* expression levels argued against this hypothesis. However, our QTL analysis suggested that *AtHKT1*, but not additional locus, control the salt adaptability of Tsu-1. Knockout of *AtHKT1* in Tsu-1 by CRISPR-Cas9 technology and complementation *athkt1-1* with Tsu-1 and Col-0 *AtHKT1* finally established that *AtHKT1* is not only a requirement for salt tolerance of Tsu-1 but is also a predominant, if not sole locus that drives the natural variation in salinity adaptation between Col-0 and Tsu-1. Though *AtHKT1* has been found to drive natural variation in leaf sodium content, this is the first direct evidence to show that *AtHKT1* plays a positive role in natural selection of saline adaptive *A*. *thaliana* accessions. These genetic evidences revealed that the Tsu-1 allele of *AtHKT1* is hyper-functional but not hypo-functional.

### The saline adaptive accession Tsu-1 employs a more efficient mechanism of *AtHKT1* for restricting sodium accumulation in flowers

Our grafting experiments revealed that the saline adaptability of Tsu-1 is driven by shoot *AtHKT1* expression, which is in contrast with the premise that salt tolerance of Col-0 is predominantly driven by root *AtHKT1* expression. This data indicates that *AtHKT1* functions differently in different accessions. GUS reporter system and qRT-PCR confirmed that Tsu-1 *AtHKT1* is hyper-functional relative to Col-0 *AtHKT1* due to its high expression in stems. Interestingly, histochemical staining experiment revealed that Tsu-1 *AtHKT1* is predominantly expressed in xylem parenchyma cells of stems, suggesting that it functions in restricting sodium in the stems. Measurements of stem and floral sodium concentrations of different genotypes confirmed this hypothesis. Therefore, the Tsu-1 *AtHKT1* functions through a new mechanism, which is to restrict sodium in the leaves and stems. This mechanism is in contrast with that of Col-0 *AtHKT1* that restricts sodium in the roots.

Flower is the most important organ of the plant. However, the germ cells in flowers are very sensitive to sodium, as such cells are weak in sodium compartmentalization and detoxification due to low vacuole capacity. By contrast, leaf cells have very large vacuoles; thus, even if they accumulate a high level of sodium, it is not highly toxic if it is sequestered in the vacuole [19]. Instead, the high accumulation of sodium in leaves would facilitate resistance to osmotic stress. This difference results in flowers being much more sensitive to salt compared to leaves. Consequently, reducing the flower sodium content but not the leaf or stem sodium content is much more important for *A*. *thaliana* upon salt stress. The Tsu-1 *AtHKT1* and the Col-0 *AtHKT1* represent two mechanisms for restricting sodium flow to flowers; Tsu-1 AtHKT1 unloads sodium to the leaves and stems, and Col-0 unloads sodium in the roots. According to the floral sodium concentrations in Tsu-1 and Col-0, we can conclude that the Tus-1 mechanism is more effective than Col-0 mechanism in limiting sodium flow to flowers. In addition, a previous study has shown that the Col-0 mechanism could inhibit root growth upon salt stress due to its improving sodium accumulation in the roots [17]. These findings together explain why the mechanism of Tsu-1 *AtHKT1* endows *A*. *thaliana* with a higher adaptation ability to saline conditions.

However, we also noticed that the Col-0 mechanism was selected by most inland accessions though it represents a low efficient mechanism in salt tolerance. So far, it is hard to conclude what is the driving force behind the selection of such a mechanism, but we suspect that it might be associated with the edaphic sodium concentrations. Inland soil contains subtoxic levels of sodium, and accumulating sodium in the roots thus might be the most economical way for *A*. *thaliana* to minimize the side-effect of sodium on flower development. By contrast, the Tsu-1 mechanism involves transporting of sodium to shoot cells and sequestrating it into the vacuoles that consume much more energy. Therefore, the selection of Col-0 mechanism or Tsu-1 mechanism might reflect the sodium levels in their habitat.

### The polymorphisms in the promoter region might be responsible for the divergence in expression between Col-0 *AtHKT1* and Tsu-1 *AtHKT1*

According to a previous study, one of the tandem repeats in the distal promoter of *AtHKT1* is an enhancer that promotes the expression of *AtHKT1* in roots, and the absence of this distal repeat in Tsu-1 was confirmed to be responsible for the down regulation of *AtHKT1* in roots [13]. However, we noted in the results of a previous study that the expression of *AtHKT1* is apparently upregulated in shoots and markedly downregulated in roots in the absence of this hypothesized distal enhancer [14]. In our study, the expression of Tsu-1 *AtHKT1* was significantly higher than that of Col-0 *AtHKT1* in the shoots of young seedlings. This observation suggested that the second tandem repeat might not only serve as an enhancer in roots but may also function as a repressor in shoots. In this case, the high expression of *AtHKT1* in Tsu-1 stems is probably caused by the deletion of the second tandem repeat. *AtHKT1* has also been reported to be regulated by DNA methylation in the promoter [14]. However, we found no difference of the small RNA target site in the promoters of *AtHKT1* between Tsu-1 and Col-0 (S4 Fig). Of course, we cannot exclude the possibility that other polymorphisms among the many in the promoter region control the variation in *AtHKT1* expression patterns. Further research is necessary to clarify this question.

### Floral sodium contents upon mild salt stress might be a better indicator of plant adaptation to saline conditions

Shoot sodium content is often used as a parameter for assessing salt tolerance of plants, but the relationship between shoot Na^+^ and salt tolerance raises arguments. In rice, low shoot Na^+^ is an indicator of salt tolerance [27]. A typical case is the identification of *SKC1*/*OsHKT1;5* that reduces leaf salt content and enhances salt tolerance [9]. However, it was reported that the growth and yield of wheat upon salt stress is not correlated with sodium concentrations in leaf blade or the whole shoot [28]. Meanwhile, the salt tolerant tomato varieties accumulate higher sodium in stems and leaves than the sensitive varieties [29,30]. These findings suggest that the relationship between shoot sodium content and salt tolerance might be genetic background dependent, and therefore not stable index for assessing plant salt tolerance. This study further supports the above conclusion. In this study, we observed that the sodium levels in flowers, but not in leaves, are positively associated with salinity adaptability. This suggests that floral sodium content might be more suitable for assessing salt tolerance of plants. Of course, this does not mean the leaf sodium does not matter for salt tolerance of plants. Actually, the increased leaf sodium causes severe leaf necrosis in *athkt1* null mutants when exposed to high salt conditions [16,18,31]. But upon mild salt stress, the flowering phenotype of *athkt1* null mutants is more obvious than the leaf phenotype, as its leaf still has photosynthesis ability (S5A Fig), but it is almost sterile in seed production. Therefore, leaf sodium may play different roles upon different levels of salt conditions. It is worthwhile pointing out that *A*. *thaliana* is a species of glycophytes and even salt tolerant ecotypes/accessions may not successfully colonize in high salt area. Even Tsu-1 barely produces seeds when exposed to 150 mM NaCl. Therefore, mild salt stress more accurately reflects the real habitat of *A*. *thaliana*. On the other hand, the leaf necrosis in Tsu-1 caused by high salt treatment is less severe than Col-0 (S5B Fig), though Tsu-1 accumulates as much sodium as *hkt1* null mutants in the leaves. This could be caused by differential expression pattern of *AtHKT1* in leaves, or controlled by some other QTLs/genes.

To our knowledge, the relationship between flower sodium content and saline adaptability has never been reported. Identifying the positive relationship between the two factors provides a new angle to assess plant salt adaptability. Of course, we are not sure if this conclusion could be extended to all plant species in current stage, and further investigation is necessary to answer this question. The seed production is a most important trait of crops in agriculture. Our finding that different expression patterns and levels of *AtHKT1* result in different yield upon salt stress could inspire engineering salt tolerant plants by manipulating expression pattern of *HKT1*. Moreover, this finding could also be translated to crops to improve their yields in saline area.

## Materials & methods

### Plant material and salt treatment

Seeds of *A*. *thaliana* were surface sterilized and sown on 1/2 Murashige and Skoog (MS, Sigma-Aldrich, St. Louis, USA) medium (pH 5.7) containing 1% (w/v) sucrose, followed by stratification at 4 °C in dark for 5 days. The seedlings were transferred into pots with artificial soil in a phytotron (16 h light/8 h dark, 22 °C day/20 °C night, 80% humidity, 80 μmol·m^-2^·s^-1^) after being grown on the plates for 7 days. Two-week-old soil-grown plants that were irrigated with 100 mM NaCl twice for two weeks and 1 additional week later plants were used for phenotyping. Four-week-old plants irrigated with 100 mM NaCl for 24 h were used for gene expression analysis and GUS staining. Each line used for salt treatment contained ≥ 12 plants, and every treatment included 3 biological replicates. The *athkt1* mutant was isolated from an F2 population derived from a backcross between *hkt1-1* (CS6531, obtained from the Arabidopsis Biological Resource Center) and Col-0.

### Hydroponic culture

Seeds of *A*. *thaliana* (Tsu-1, Col-0, *AtHKT1* RNAi lines and *athkt1*) were stratified for 5 days at 4 °C in water. The stratified seeds were sowed in punctured holes of 1.5-mL Eppendorf tube caps stuffed with 0.1% agar, and the caps were then placed on 1.5-mL Eppendorf tube containing Hoagland solution for 14 days in a chamber at 22 °C, 70% relative humidity, and light intensity of 80 μmol·m^-2^· s^-1^ on 8 h light/16 h dark. The seedlings together with caps were then transferred to a big box containing Hoagland solution for growing 2 additional weeks. The culture solution was refreshed every 5 days. The roots of one-month-old seedlings were collected for qRT analysis.

### QTL mapping analysis

An F2 population derived from Tsu-1 and *athkt1* was used to map the causal gene of the salt tolerance of Tsu-1. The number of seeds was calculated based on an estimation of the total grain weight and 100-grain weight. A set of 36 CAPS (Cleaved Amplified Polymorphism Sequences) and SSR (Simple Sequence Repeat) markers covering the 5 chromosomes of *A*. *thaliana* were used to genotype all F2 individuals. QTL analysis was performed using the R/qtl package (http://www.rqtl.org). To narrow down the candidate region, 537 F2 individuals and 10 newly developed CAPS markers were used to fine map the target region. All CAPS and SSR markers are listed in S2 Table.

### Vector construction and plant transformation

For the complementation experiment, a 9.3-kb *AtHKT1* fragment including a 5.3-kb promoter region from Col-0 was amplified by overlap PCR using primers HKT-pro-gene L, PHMS-HKT1-MID-R, PHMS-HKT1-MID-L and HKT-pro-gene R (S3 Table). The complementation fragment for Tsu-1 *AtHKT1* was amplified using the same strategy but with primers Tsu-HKT-pro-gene L, PHMS-HKT1-MID-R, PHMS-HKT1-MID-L and HKT-pro-gene R. The fragments were cloned into a binary vector, pHMS, which was modified from the pHB vector [32] by infusion recombination cloning using a Hieff Clone One-step PCR Cloning Kit (Yisheng Co. Ltm, Shanghai, China).

To construct the *promoter*:*GUS* vector, the *AtHKT1* promoters were amplified from genomic DNA using primers HKT-GUSL TONG and HKT-GUSR TONG for Col-0 and primers HKT-GUSL TONG 2 and HKT-GUSR TONG for Tsu-1. The fragments were recombined into the binary vector pCAMBIA1303 using the Hieff Clone One-step PCR Cloning Kit. GUS histochemical staining was performed as described previously [9].

To construct the Col-0 *AtHKT1* RNAi vector, two 184-bp fragments from the *AtHKT1* full-length cDNA sequence were amplified using the primers *AtHKT1 F (Pst*I restriction site) and R (*Xba*I restriction site) for sense and *AtHKT1F* (*Sac*I restriction site) and *R* (*Not*I restriction site) for antisense. Both amplified fragments were sequenced and inserted into an intermediate vector, pBluescript II SK (+/-), containing a 120-nucleotide intron from the *A*. *thaliana* RTM1 gene at the *Xba*I and *Not*I sites. Then, the hairpin fragment was sub-cloned into the binary vector pCAMBIA2301, which contained a cauliflower mosaic virus (CaMV) 35S promoter and a NOS terminator. The kanamycin resistance gene (*NPT II*) was used as a selective marker gene.

The construction of a CRISPR-Cas9 vector targeting *AtHKT1* was performed as described previously [22]. Two oligos, *AtHKT1* sgRNA4-F and sgRNA4-R, were synthesized and annealed to form a target site of 20bp in length with a requisite proto-spacer-adjacent motif, PAM (NGG) sequence at the 3’ end and G at the 5’ end of the sequence. The target site, containing two *Bbs*I digestion sites, was cloned into the intermediate vector AtU6-26SK. The chimeric RNA expression cassette between *Kpn*I and *Sal*I was cloned into the *Kpn*I and *EcoR*I sites of the pCambia1300 vector (Cambia, Canberra, Australia) together with the *Sal*I and *EcoR*I fragment of the Cas9 expression cassette from the 35S-Cas9-SK vector, which was a gift from the Zhu lab [22].

The expression constructs were transformed into *Agrobacterium tumefaciens* strain GV3101, and were then introduced into the backgrounds as indicated using the floral dip method (26). Transgenic lines were screened on 1/2 MS medium solidified with agar containing 50 mg/L hygromycin and 1% sucrose.

### Electrophysiological analysis of AtHKT1 in *X*. *laevis* oocytes

We inserted the cDNA of *AtHKT1* from the Col-0 and Tsu-1 alleles into the *Sma*I site of the expression vector pGEMHE. The capped cRNA was transcribed using the RiboMAX Large Scale RNA Production System-T7 kit (P1300, Promega, Wisconsin, USA). The cRNA quality was verified using agarose gel electrophoresis. The concentration was determined at 260 and 280 nm and adjusted to a final concentration of 0.8 μg μl^-1^. We injected freshly isolated *X*. *laevis* oocytes with 23 nL of cRNA and used the oocytes for voltage-clamp experiments 3–4 days later. Electrophysiological experiments were conducted using a two-electrode voltage clamp amplifier as described previously [9]. We bathed the oocytes in a solution containing 6 mM MgCl_2_, 1.8 mM CaCl_2_, 10 mM MES-Tris (pH 5.5), 185 mM D-mannitol and 10 mM Na-glutamate salts. The voltage protocols used are described in the figure legends.

### ICP-MS analysis

The process of *A*. *thaliana* elemental analysis via inductively coupled plasma mass spectrometry (ICP-MS) has been described previously [33]. Briefly, different tissues, including flowers, whole stems and rosette leaves, from five-week-old plants treated twice with 100 mM NaCl or from untreated plants were cut with a scalpel while holding the plants with plastic tweezers. The collected tissues were rinsed with four times with 18-MΩ water in a 1000-mL breaker to wash off the impurities. Then, the rinsed samples were placed into a glass tube, and the samples were shifted to the bottom of the tube, making sure that no samples were left on the tube wall. The tubes were transferred to an oven at 65°C for 12 h. After cooling, twelve samples were weighed out on an analytical balance. All the samples, including the blank controls, were digested with 1 mL of concentrated nitric acid containing an indium (In) internal standard at 115°Cfor 4 h, and then the digested samples were diluted to 10 mL with 18 MΩ water. Elemental analysis for Li, B, Na, Mg, P, S, K, Ca, Mn, Fe, Co, Ni, Cu, Zn, As, Se, Rb, Sr, Mo, and Cd was performed using an ICP-MS (NexION 350D; PerkinElmer) coupled to an Apex desolvation system and an SC-4 DX auto sampler (Elemental Scientific Inc., Omaha, NE, US). All samples were normalized to calculate weight, as determined using a heuristic algorithm using the best-measured elements, the weights of the eight weighed samples, and the solution concentrations, as previously described.

### Grafting of *A*. *thaliana* plants

Seedlings were grafted as previously described [13]. Graft unions were examined under a stereoscope before transfer to potting soil to identify any adventitious root formation at or above the graft unions. Healthy grafted plants were transferred to pots and grown in a controlled environment for 2 weeks. Then, the plants were irrigated with a 100 mM NaCl solution twice, and after 2 weeks, the plants were photographed. After harvesting, the graft unions were examined again, and grafted plants with adventitious roots or without a clear graft union were excluded from subsequent analysis.

### qRT-PCR

Total RNA was extracted from plants using TRNzol A+ RNA Purification reagent (TIANGEN, DP421, Beijing, China). Two micrograms of total RNA were used to synthesize first-strand cDNA with TransScript One-Step gDNA Removal and cDNA Synthesis SuperMix (Transgen, AT311-02, Beijing, China). qRT-PCR was performed using SYBR Green PCR Master Mix (TRT-101, TOYOBO, Osaka, Japan) with the first-strand cDNA as a template on a Real-Time PCR System (Bio-Rad CFX thermocycler, California, USA). Primers for qRT-PCR were designed using Primer Express Software Version 3.0 (Applied Biosystems, USA). The primers HKT1 exon2-3-1L and HKT1 exon2-3-1R were designed to span an exon-exon junction and were used to detect the gene expression level. The primers UBCF and UBCR were designed for *UBIQUITIN-CONJUGATING ENZYME21* (At5g25760), which was used as the control gene. The primer sequences are shown in S3 Table. Expression data analysis was performed as described previously [34].

## Supporting information

S1 Fig Targeted indel mutations induced by engineered sgRNA:Cas9 at the *AtHKT1* gene in Tsu-1. (A) A thymine insertion between the 450^th^ and 451^st^ nucleotides of *AtHKT1* coding sequence. (B) Four-nucleotide deletion between the 447^th^and 450^th^of *AtHKT1* coding sequence shown in red box. Sequences shown were amplified from genomic DNA and sequenced after cloned into vectors. (C) Phenotypes of Tsu-1, *hkt1-c1* and *hkt1-c2* under normal conditions. (D) Phenotypes of Tsu-1, *hkt1-c1* and *hkt1-c2* under salt stress condition. Five-week-old plants irrigated without or twice with 100 mM NaCl were taken photographed.
(TIF)

S2 Fig Allelism test of *hkt1-c1*, *hkt1-c2* and *athkt1*. (A) F1 plants derived from a cross between *athkt1* and *hkt1-c1* under salt stress condition. (B) F1 plants derived from a cross between *athkt1* and*hkt1-c2* under salt stress condition. Five-week-old plants were irrigated twice with 100 mM NaCl.
(TIFF)

S3 Fig **Expression of *AtHKT1* in rosette leaves (A) and flowers (B) in Col-0 and Tsu-1 revealed by qRT-PCR.** Four-week-old plants before and after treatment with 100 mM NaCl for 24 h. Data represented with mean ±SE, *n* = 6.
(TIFF)

S4 Fig Polymorphisms of *AtHKT1* between Col-0and Tsu-1. (A) Major large indel polymorphisms of *AtHKT1* between Col-0 and Tsu-1. (B) Detailed polymorphisms of Tsu-1 *AtHKT1* compared to Col-0 *AtHKT1*.
(TIFF)

S5 Fig Photosynthetic changes of Col-0, *athkt1* and Tsu-1 when exposed to salt stress. Four-week-old plants before and after treatment with 100 mM NaCl for 3 days. Fv/Fm, an indicator for light-adapted maximum quantum yield of PSII, was measured at 0 day, 1 day, 2 day and 3 day post-salt treatment.
(TIFF)

S1 Table List of genes in the 100-kb candidate region between genetic markers GM635 and GM645. (DOCX)

S2 Table Primers used for QTL mapping. (DOCX)

S3 Table Primers used for sequencing and vector construction. (DOCX)

## References

[1] Ma Y, Dai X, Xu Y, Luo W, Zheng X, Zeng D, et al COLD1 confers chilling tolerance in rice. Cell. 2015;160: 1209–1221. doi: 10.1016/j.cell.2015.01.046 25728666 10.1016/j.cell.2015.01.046

[2] Li Y, Huang Y, Bergelson J, Nordborg M, Borevitz JO. Association mapping of local climate-sensitive quantitative trait loci in Arabidopsis thaliana. Proc Natl Acad Sci USA. 2010;107: 21199–21204. doi: 10.1073/pnas.1007431107 21078970 10.1073/pnas.1007431107 PMC3000268

[3] Lu S, Zhao X, Hu Y, Liu S, Nan H, Li X, et al Natural variation at the soybean J locus improves adaptation to the tropics and enhances yield. Nat Genet. 2017;49: 773–779. doi: 10.1038/ng.3819 28319089 10.1038/ng.3819

[4] Todesco M, Balasubramanian S, Hu TT, Traw MB, Horton M, Epple P, et al Natural allelic variation underlying a major fitness trade-off in Arabidopsis thaliana. Nature. 2010;465: 632–636. doi: 10.1038/nature09083 20520716 10.1038/nature09083 PMC3055268

[5] Chao D-Y, Dilkes B, Luo H, Douglas A, Yakubova E, Lahner B, et al Polyploids exhibit higher potassium uptake and salinity tolerance in Arabidopsis. Science. 2013;341: 658–659. doi: 10.1126/science.1240561 23887874 10.1126/science.1240561 PMC4018534

[6] Rubio F, Gassmann W, Schroeder JI. Sodium-driven potassium uptake by the plant potassium transporter HKT1 and mutations conferring salt tolerance. Science. 1995;270: 1660–1663. 7502075 10.1126/science.270.5242.1660

[7] Uozumi N, Kim EJ, Rubio F, Yamaguchi T, Muto S, Tsuboi A, et al The Arabidopsis HKT1 gene homolog mediates inward Na(+) currents in xenopus laevis oocytes and Na(+) uptake in Saccharomyces cerevisiae. Plant Physiol. 2000;122: 1249–1259. 10759522 10.1104/pp.122.4.1249 PMC58961

[8] Sunarpi, Horie T, Motoda J, Kubo M, Yang H, Yoda K, et al Enhanced salt tolerance mediated by AtHKT1 transporter-induced Na unloading from xylem vessels to xylem parenchyma cells. Plant J. 2005;44: 928–938. doi: 10.1111/j.1365-313X.2005.02595.x 16359386 10.1111/j.1365-313X.2005.02595.x

[9] Ren Z-H, Gao J-P, Li L-G, Cai X-L, Huang W, Chao D-Y, et al A rice quantitative trait locus for salt tolerance encodes a sodium transporter. Nat Genet. 2005;37: 1141–1146. doi: 10.1038/ng1643 16155566 10.1038/ng1643

[10] Kobayashi NI, Yamaji N, Yamamoto H, Okubo K, Ueno H, Costa A, et al OsHKT1;5 mediates Na(+) exclusion in the vasculature to protect leaf blades and reproductive tissues from salt toxicity in rice. Plant J. 3rd ed. 2017;143: 1918 doi: 10.1111/tpj.13595 10.1111/tpj.13595 28488420

[11] DAVENPORT RJ, MUÑOZ-MAYOR A, Jha D, ESSAH PA, Rus A, Tester M. The Na +transporter AtHKT1;1 controls retrieval of Na +from the xylem in Arabidopsis. Plant Cell Environ. 2007;30: 497–507. doi: 10.1111/j.1365-3040.2007.01637.x 17324235 10.1111/j.1365-3040.2007.01637.x

[12] Busoms S, Teres J, Huang X-Y, Bomblies K, Danku J, Douglas A, et al Salinity Is an Agent of Divergent Selection Driving Local Adaptation of Arabidopsis to Coastal Habitats. Plant Physiol. 2015;168: 915–929. doi: 10.1104/pp.15.00427 26034264 10.1104/pp.15.00427 PMC4741335

[13] Rus A, Baxter I, Muthukumar B, Gustin J, Lahner B, Yakubova E, et al Natural variants of AtHKT1 enhance Na+ accumulation in two wild populations of Arabidopsis. PLoS Genet. 2006;2: e210 doi: 10.1371/journal.pgen.0020210 17140289 10.1371/journal.pgen.0020210 PMC1665649

[14] Baek D, Jiang J, Chung J-S, Wang B, Chen J, Xin Z, et al Regulated AtHKT1 gene expression by a distal enhancer element and DNA methylation in the promoter plays an important role in salt tolerance. Plant Cell Physiol. 2011;52: 149–161. doi: 10.1093/pcp/pcq182 21097475 10.1093/pcp/pcq182

[15] Baxter I, Brazelton JN, Yu D, Huang YS, Lahner B, Yakubova E, et al A Coastal Cline in Sodium Accumulation in Arabidopsis thaliana Is Driven by Natural Variation of the Sodium Transporter AtHKT1;1. Copenhaver GP, editor. PLoS Genet. 2010;6: e1001193 doi: 10.1371/journal.pgen.1001193 21085628 10.1371/journal.pgen.1001193 PMC2978683

[16] Berthomieu P, Conéjéro G, Nublat A, Brackenbury WJ, Lambert C, Savio C, et al Functional analysis of AtHKT1 in Arabidopsis shows that Na(+) recirculation by the phloem is crucial for salt tolerance. EMBO J. 2003;22: 2004–2014. doi: 10.1093/emboj/cdg207 12727868 10.1093/emboj/cdg207 PMC156079

[17] Rus A. AtHKT1 Facilitates Na+ Homeostasis and K+ Nutrition in Planta. Plant Physiol. 2004;136: 2500–2511. doi: 10.1104/pp.104.042234 15347798 10.1104/pp.104.042234 PMC523317

[18] Horie T, Horie R, Chan W-Y, Leung H-Y, Schroeder JI. Calcium regulation of sodium hypersensitivities of sos3 and athkt1 mutants. Plant Cell Physiol. 2006;47: 622–633. doi: 10.1093/pcp/pcj029 16540484 10.1093/pcp/pcj029

[19] Munns R, Tester M. Mechanisms of salinity tolerance. Annu Rev Plant Biol. 2008;59: 651–681. doi: 10.1146/annurev.arplant.59.032607.092911 18444910 10.1146/annurev.arplant.59.032607.092911

[20] Munns R, James RA, Xu B, Athman A, Conn SJ, Jordans C, et al Wheat grain yield on saline soils is improved by an ancestral Na^+^ transporter gene. Nat Biotechnol. 2012;30: 360–364. doi: 10.1038/nbt.2120 22407351 10.1038/nbt.2120

[21] Rodríguez-Rosales MP, Gálvez FJ, Huertas R, Aranda MN, Baghour M, Cagnac O, et al Plant NHX cation/proton antiporters. Plant Signal Behav. 2009;4: 265–276. 19794841 10.4161/psb.4.4.7919 PMC2664485

[22] Feng Z, Zhang B, Ding W, Liu X, Yang D-L, Wei P, et al Efficient genome editing in plants using a CRISPR/Cas system. Cell Res. 2013;23: 1229–1232. doi: 10.1038/cr.2013.114 23958582 10.1038/cr.2013.114 PMC3790235

[23] Mäser P, Hosoo Y, Goshima S, Horie T, Eckelman B, Yamada K, et al Glycine residues in potassium channel-like selectivity filters determine potassium selectivity in four-loop-per-subunit HKT transporters from plants. Proc Natl Acad Sci USA. 2002;99: 6428–6433. doi: 10.1073/pnas.082123799 11959905 10.1073/pnas.082123799 PMC122965

[24] Ali A, Raddatz N, Aman R, Kim S, Park HC, Jan M, et al A Single Amino-Acid Substitution in the Sodium Transporter HKT1 Associated with Plant Salt Tolerance. Plant Physiol. 2016;171: 2112–2126. doi: 10.1104/pp.16.00569 27208305 10.1104/pp.16.00569 PMC4936583

[25] Oomen RJFJ, Benito B, Sentenac H, Rodríguez-Navarro A, Talón M, Véry A-A, et al HKT2;2/1, a K^+^-permeable transporter identified in a salt-tolerant rice cultivar through surveys of natural genetic polymorphism. Plant J. 2012;71: 750–762. doi: 10.1111/j.1365-313X.2012.05031.x 22530609 10.1111/j.1365-313X.2012.05031.x

[26] Mishra S, Singh B, Panda K, Singh BP, Singh N, Misra P, et al Association of SNP Haplotypes of HKT Family Genes with Salt Tolerance in Indian Wild Rice Germplasm. Rice (N Y). 2016;9: 15 doi: 10.1186/s12284-016-0083-8 27025598 10.1186/s12284-016-0083-8 PMC4811800

[27] Lin HX, Zhu MZ, Yano M, Gao JP, Liang ZW, Su WA, et al QTLs for Na + and K + uptake of the shoots and roots controlling rice salt tolerance. Theor Appl Genet. 2004;108: 253–260. doi: 10.1007/s00122-003-1421-y 14513218 10.1007/s00122-003-1421-y

[28] Genc Y, McDonald GK, Tester M. Reassessment of tissue Na(+) concentration as a criterion for salinity tolerance in bread wheat. Plant Cell Environ. 2007;30: 1486–1498. doi: 10.1111/j.1365-3040.2007.01726.x 17897418 10.1111/j.1365-3040.2007.01726.x

[29] Jaime-Pérez N, Pineda B, García-Sogo B, Atares A, Athman A, Byrt CS, et al The sodium transporter encoded by the HKT1;2 gene modulates sodium/potassium homeostasis in tomato shoots under salinity. Plant Cell Environ. 2017;40: 658–671. doi: 10.1111/pce.12883 27987209 10.1111/pce.12883

[30] Cuartero J, Bolarín MC, Asíns MJ, Moreno V. Increasing salt tolerance in the tomato. J Exp Bot. 2006;57: 1045–1058. doi: 10.1093/jxb/erj102 16520333 10.1093/jxb/erj102

[31] Mäser P, Eckelman B, Vaidyanathan R, Horie T, Fairbairn DJ, Kubo M, et al Altered shoot/root Na+ distribution and bifurcating salt sensitivity in Arabidopsis by genetic disruption of the Na+ transporter AtHKT1. FEBS Lett. 2002;531: 157–161. 12417304 10.1016/s0014-5793(02)03488-9

[32] Mao J, Zhang Y-C, Sang Y, Li Q-H, Yang H-Q. From The Cover: A role for Arabidopsis cryptochromes and COP1 in the regulation of stomatal opening. Proc Natl Acad Sci USA. 2005;102: 12270–12275. doi: 10.1073/pnas.0501011102 16093319 10.1073/pnas.0501011102 PMC1189306

[33] Chao D-Y, Chen Y, Chen J, Shi S, Chen Z, Wang C, et al Genome-wide association mapping identifies a new arsenate reductase enzyme critical for limiting arsenic accumulation in plants. Maloof JN, editor. PLoS Biol. 2014;12: e1002009 doi: 10.1371/journal.pbio.1002009 25464340 10.1371/journal.pbio.1002009 PMC4251824

[34] Livak KJ, Schmittgen TD. Analysis of Relative Gene Expression Data Using Real-Time Quantitative PCR and the 2−ΔΔCT Method. Methods. 2001;25: 402–408. doi: 10.1006/meth.2001.1262 11846609 10.1006/meth.2001.1262

