# Supplementary figures and images for: AtHKT1 drives adaptation of Arabidopsis thaliana to salinity by reducing floral sodium content
Source: PLoS Genet. 2017 Oct 30;13(10):e1007086. doi: 10.1371/journal.pgen.1007086 (PMC5679648; doi:10.1371/journal.pgen.1007086)

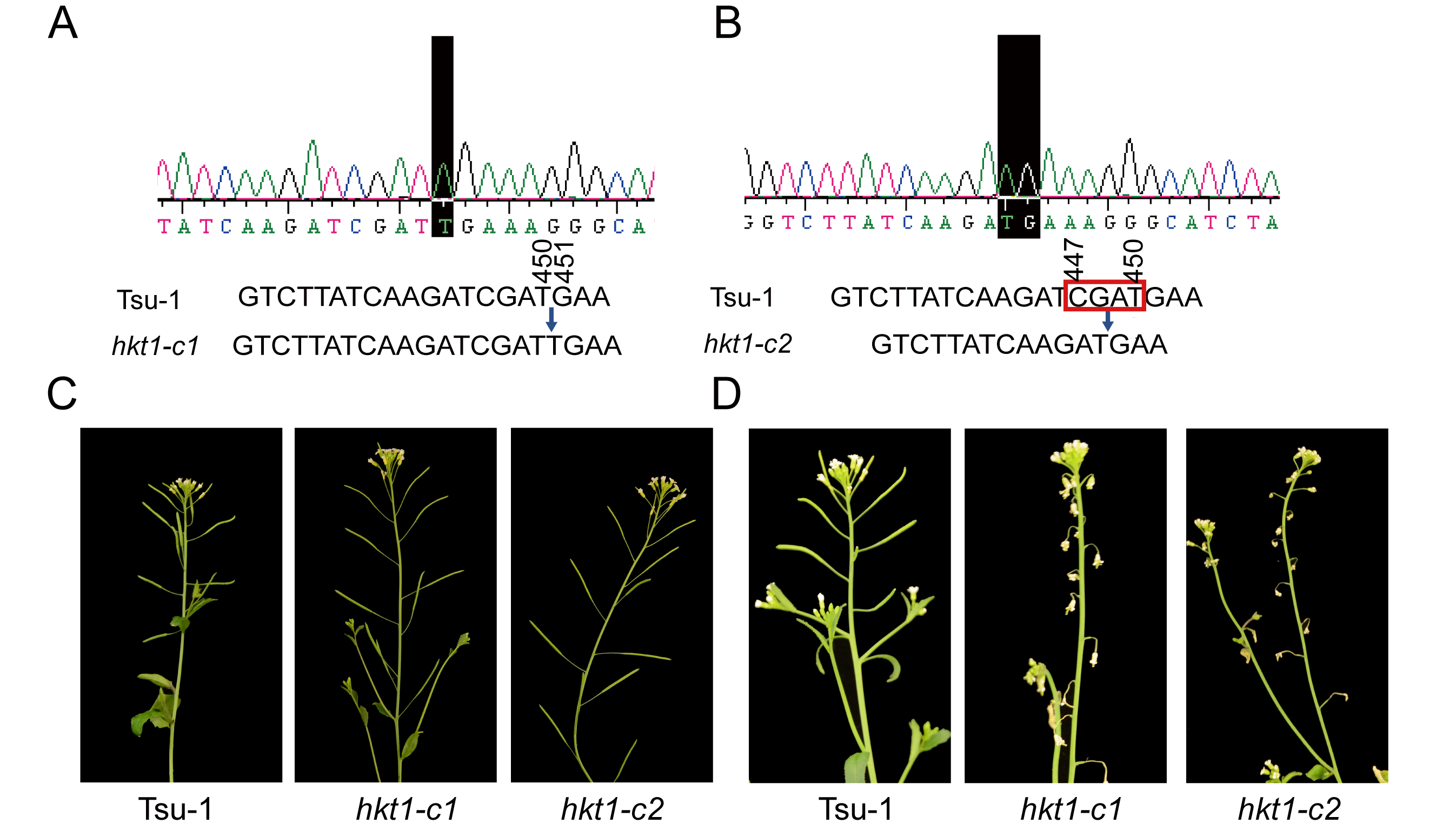

Supplement: S1 Fig — (A) A thymine insertion between the 450th and 451st nucleotides of AtHKT1 coding sequence. (B) Four-nucleotide deletion between the 447thand 450thof AtHKT1 coding sequence shown in red box. Sequences shown were amplified from genomic DNA and sequenced after cloned into vectors. (C) Phenotypes of Tsu-1, hkt1-c1 and hkt1-c2 under normal conditions. (D) Phenotypes of Tsu-1, hkt1-c1 and hkt1-c2 under salt stress condition. Five-week-old plants irrigated without or twice with 100 mM NaCl were taken photographed. (TIF) [file pgen.1007086.s001.tif]

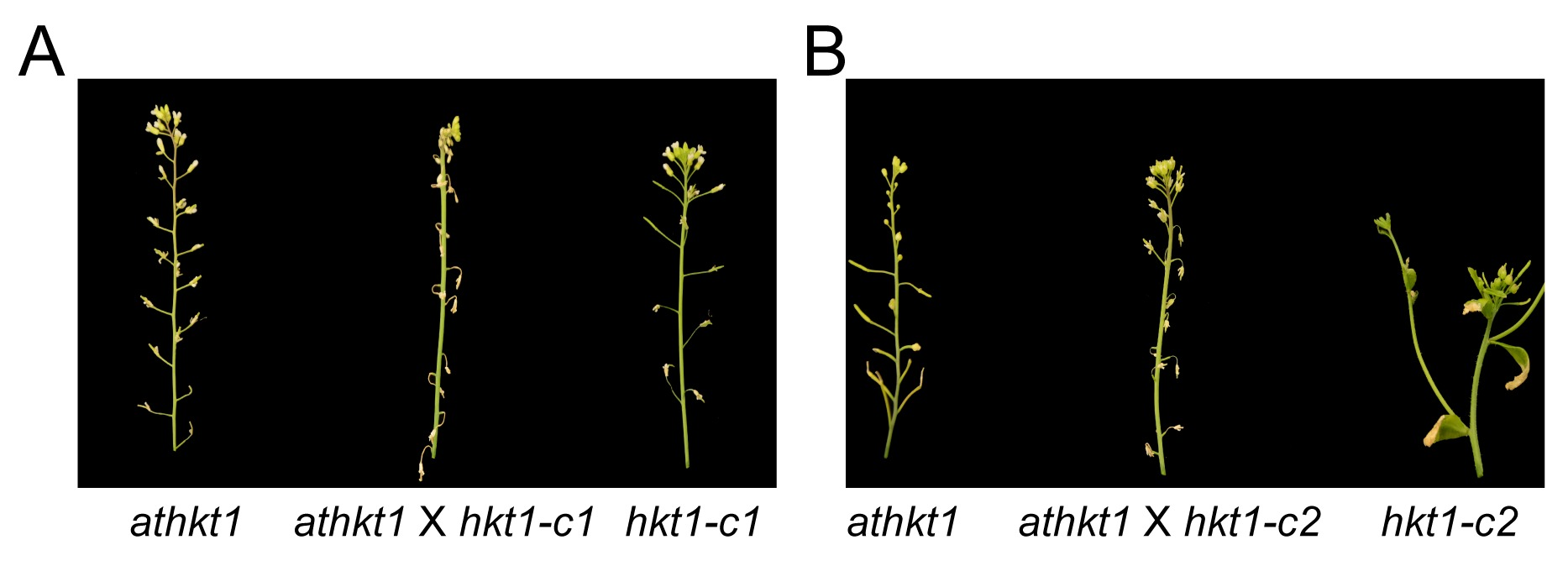

Supplement: S2 Fig — (A) F1 plants derived from a cross between athkt1 and hkt1-c1 under salt stress condition. (B) F1 plants derived from a cross between athkt1 andhkt1-c2 under salt stress condition. Five-week-old plants were irrigated twice with 100 mM NaCl. (TIFF) [file pgen.1007086.s002.tiff]

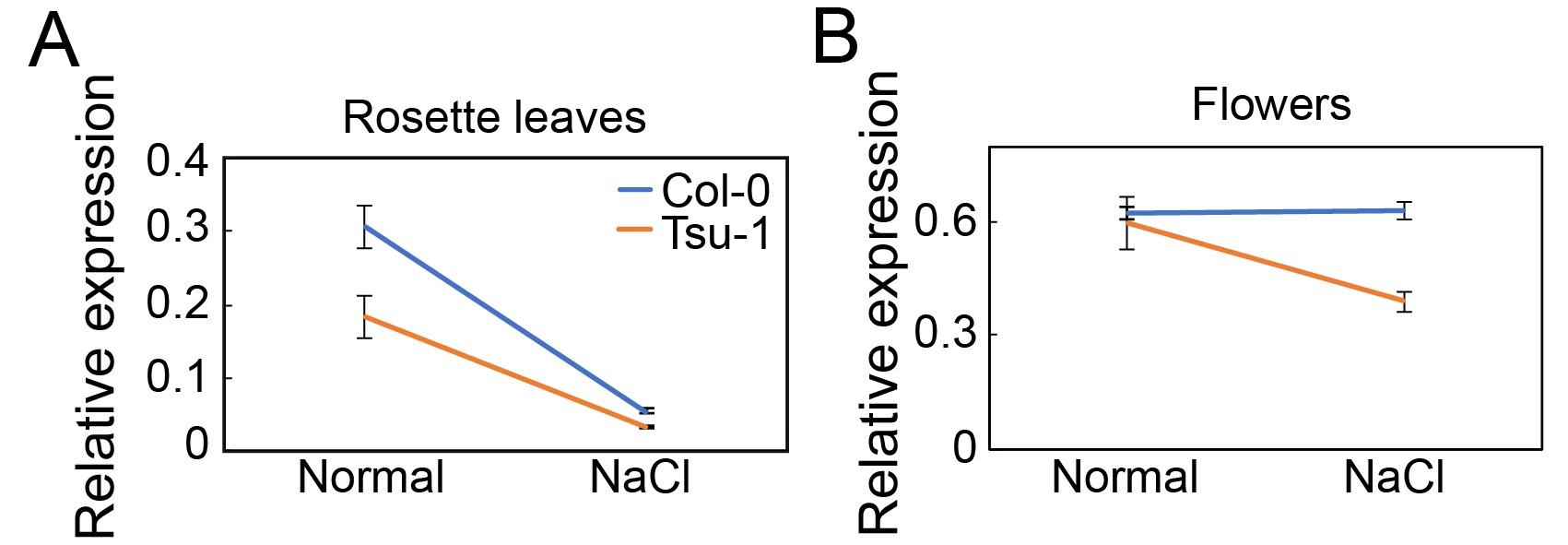

Supplement: S3 Fig — Expression of AtHKT1 in rosette leaves (A) and flowers (B) in Col-0 and Tsu-1 revealed by qRT-PCR. Four-week-old plants before and after treatment with 100 mM NaCl for 24 h. Data represented with mean ±SE, n = 6. (TIFF) [file pgen.1007086.s003.tiff]

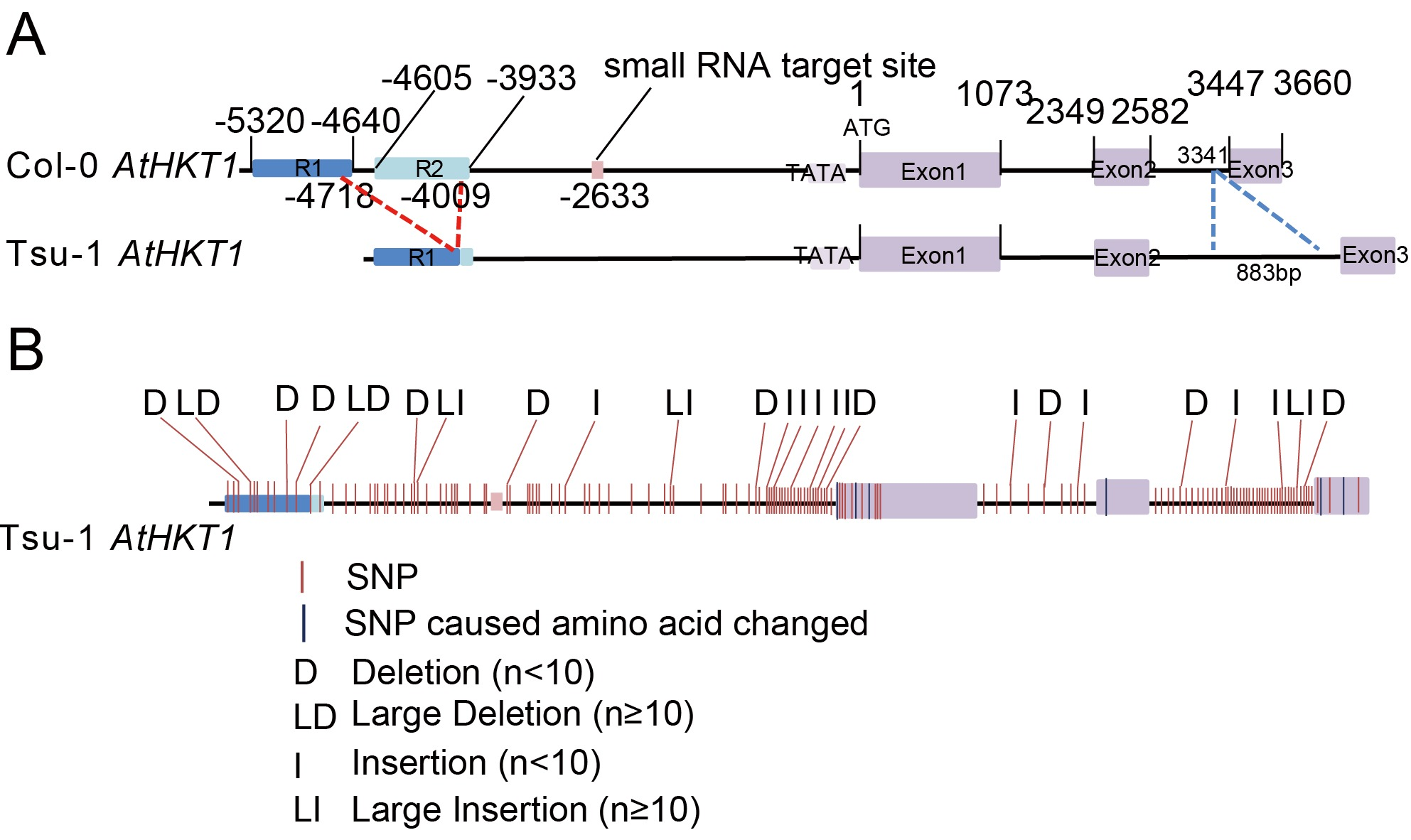

Supplement: S4 Fig — (A) Major large indel polymorphisms of AtHKT1 between Col-0 and Tsu-1. (B) Detailed polymorphisms of Tsu-1 AtHKT1 compared to Col-0 AtHKT1. (TIFF) [file pgen.1007086.s004.tiff]

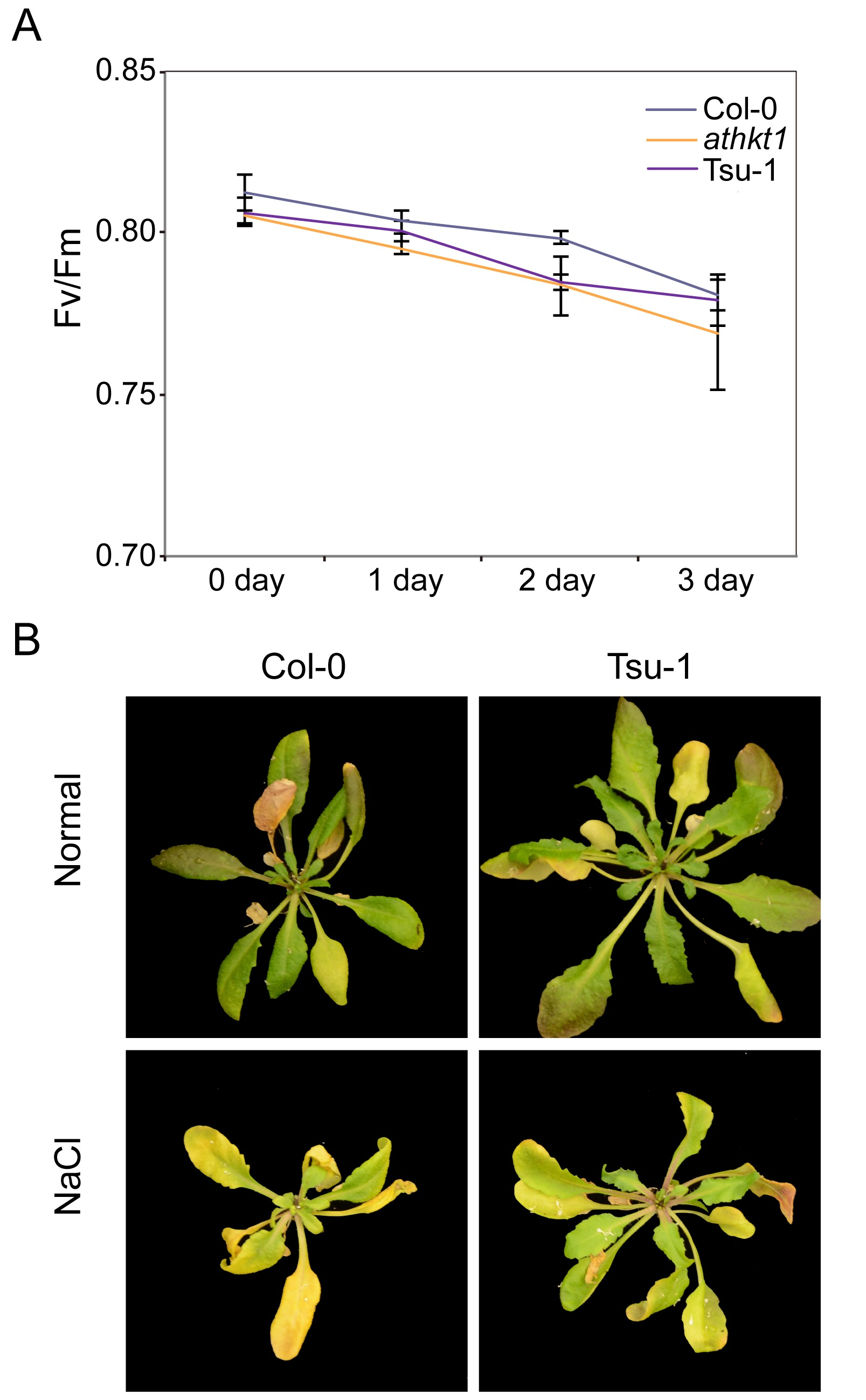

Supplement: S5 Fig — Four-week-old plants before and after treatment with 100 mM NaCl for 3 days. Fv/Fm, an indicator for light-adapted maximum quantum yield of PSII, was measured at 0 day, 1 day, 2 day and 3 day post-salt treatment. (TIFF) [file pgen.1007086.s005.tiff]
