# Supplementary material for: AtHKT1 drives adaptation of Arabidopsis thaliana to salinity by reducing floral sodium content
Source: PLoS Genet. 2017 Oct 30;13(10):e1007086. doi: 10.1371/journal.pgen.1007086 (PMC5679648; doi:10.1371/journal.pgen.1007086)
Supplement: S1 Table — (DOCX) [file pgen.1007086.s006.docx]

| **S1 Table.** List of genes in the 100-kb candidate region between genetic markers GM635 and GM645. |  |
| --- | --- |
| \| **Gene** \| **Annotation** \| \| --- \| --- \| \| AT4G10190 \| F-box and associated interaction domains-containing protein \| \| AT4G10200 \| TTF-type zinc finger protein with HAT dimerisation domain \| \| AT4G10201 \| Pseudogene of AT3G21130 \| \| AT4G10210 \| Protein of Unknown Function (DUF239) \| \| AT4G10220 \| Protein of Unknown Function (DUF239) \| \| AT4G10230 \| Unknown protein \| \| AT4G10240 \| B-BOX DOMAIN PROTEIN 23 \| \| AT4G10250 \| Columbia endomembrane-localized small heat shock protein \| \| AT4G10260 \| pfkB-like carbohydrate kinase family protein \| \| AT4G10265 \| Wound-responsive family protein \| \| AT4G10270 \| Wound-responsive family protein \| \| AT4G10280 \| RmlC-like cupins superfamily protein \| \| AT4G10290 \| RmlC-like cupins superfamily protein \| \| AT4G10300 \| RmlC-like cupins superfamily protein \| \| AT4G10310 \| A sodium transporter (HKT1) expressed in xylem parenchyma cells \| \| AT4G10320 \| tRNA synthetase class I (I, L, M and V) family protein \| \| AT4G10330 \| Glycine-rich protein \| \| AT4G10340 \| Photosystem II encoding the light-harvesting chlorophyll a/b binding protein \| \| AT4G10345 \| Encodes a microRNA of unknown function \| \| AT4G10350 \| Encode a transcription factor \| \| AT4G10360 \| TRAM, LAG1 and CLN8 (TLC) lipid-sensing domain containing protein \| \| AT4G10370 \| Cysteine/Histidine-rich C1 domain family protein \| \| AT4G10380 \| NOD26-LIKE INTRINSIC PROTEIN 5;1, Boric acid channel. \| \| AT4G10390 \| Protein kinase superfamily protein \| \| AT4G10400 \| F-box/RNI-like/FBD-like domains-containing protein \| \| AT4G10410 \| Leucine-rich repeat (LRR) family protein \| |  |
|  |  |
