# Supplementary material for: AtHKT1 drives adaptation of Arabidopsis thaliana to salinity by reducing floral sodium content
Source: PLoS Genet. 2017 Oct 30;13(10):e1007086. doi: 10.1371/journal.pgen.1007086 (PMC5679648; doi:10.1371/journal.pgen.1007086)
Supplement: S2 Table — (DOCX) [file pgen.1007086.s007.docx]

|  | | | | | | |  |
| --- | --- | --- | --- | --- | --- | --- | --- |
| **S2 Table.** Primers used for QTL mapping. | | | | | |  |  |
| **Name** | **Sequence** | **Chromosome** | **Site** | **Marker** | **Enzyme** |  |  |
| T7I23 | CATGCACGTACGATTTGTTTAAC | chr1 | W/427343-427365 | SSR |  |  |  |
|  | GTGTCCTTTTTTCTCAACGATG | chr1 | C/427521-427542 | SSR |  |  |  |
| 1C3M | TAAATTGTGGAAGGGCCAGT | chr1 | 3073052 | SSR |  |  |  |
|  | CCGCACAATTCTCTCAATCC | chr1 | 3073135 | SSR |  |  |  |
| T-1C8MU | TGCCTGCTATACAACTTAACAGTTTC | chr1 | 8M | SSR |  |  |  |
| T-1C8ML | CGTCGTCTAGAAAATACCAATGA | chr1 |  | SSR |  |  |  |
| UPSC_1-13122 | ATCAAAGGTTGCCACAAATG | chr1 | 13.122M | SSR |  |  |  |
|  | TTCGGTTTGATTTGGGTTTC | chr1 |  | SSR |  |  |  |
| 1C17.02-F | AAAGTGGAGATTTCTCCCGATA | chr1 | 17.02 | SSR |  |  |  |
| 1C17.02-R | GAAATTTGAAACGGAAATTAG | chr1 |  | SSR |  |  |  |
| 1C22M | GCTTTTGGGCTCTTGAGTTG | chr1 | 22077469 | SSR |  |  |  |
|  | CCAAAGAATACCCACGAGTGA | chr1 |  | SSR |  |  |  |
| ATHATPASE | CTGGGAACGGTTCGATTCGAGC | chr1 | C/28533900-28533921 | SSR |  |  |  |
|  | GTTCACAGAGAGACTCATAAACCA | chr1 |  | SSR |  |  |  |
| 2C5M | TGGACGTTAGCGAGATGTTG | chr2 | 5612662 | SSR |  |  |  |
|  | AGAAAGAAGTGACCGTAAAATGG | chr2 | 5612780 | SSR |  |  |  |
| MSAT2.36 | GATCTGCCTCTTGATCAGC | chr2 | W/8678440-8678458 | SSR |  |  |  |
|  | CCAAGAACTCAAAACCGTT | chr2 | C/8678579-8678597 | SSR |  |  |  |
| 2C11M | AGAGAGGTGCGCTTGTTGAT | chr2 | 11443140 | SSR |  |  |  |
|  | TCATTAGAAATCGATCCCACA | chr2 | 11443221 | SSR |  |  |  |
| MSAT2.4 | TGGGTTTTTGTGGGTC | chr2 | W/13824525-13824540 | SSR |  |  |  |
|  | GTATTATTGTGCTGCCTTTT | chr2 | C/13824793-13824812 | SSR |  |  |  |
| MSAT2.9 | TAAAAGAGTCCCTCGTAAAG | chr2 | C/16585397-16585417 | SSR |  |  |  |
|  | GTTGTTGTTGTGGCATT | chr2 |  | SSR |  |  |  |
| 2C18M | AGAGGACATGTATTGGCAGTG | chr2 | 18154108 | SSR |  |  |  |
|  | TTGGAGAAGGTCCCCAAAATG | chr2 | 18154156 | SSR |  |  |  |
| 3C3MU | GGATGATTACGGTTTCTACTC | chr3 | 3M | SSR |  |  |  |
| 3C3ML | CCAGTTCTCAGGCTTCTTC | chr3 |  | SSR |  |  |  |
| 3C4M | AAACGGCCAAAACATAATGG | chr3 | 4124144 | SSR |  |  |  |
|  | TTGTCCTCCAACGGATAAGC | chr3 |  | SSR |  |  |  |
| 3C6M | GCGGCTTTCAGTCTTGTTTC | chr3 | 5984119 | SSR |  |  |  |
|  | TGTCATCACTATCATCGGGTACA | chr3 |  | SSR |  |  |  |
| 3C11M | ACAAAATGGCTGCAAAAATG | chr3 | 11287477 | SSR |  |  |  |
|  | TTGGTTGAAATTAGGCTCGTT | chr3 |  | SSR |  |  |  |
| 3C16M | TTGTTTTCCTTGTGCTGAGGT | chr3 | 16099073 | SSR |  |  |  |
|  | CAAACTTGAAAACTTCAAACTCCA | chr3 |  | SSR |  |  |  |
| 3C20M | CAATTTCCTTTGGGTTTGCT | chr3 | 20016732 | SSR |  |  |  |
|  | ATTCGGATTGTGAGGTGGAC | chr3 |  | SSR |  |  |  |
| NGA6 | TGGATTTCTTCCTCTCTTCAC | chr3 | C/23031172-23031192 | SSR |  |  |  |
|  | ATGGAGAAGCTTACACTGATC | chr3 |  | SSR |  |  |  |
| T-4C0.6MU-1 | GGTTTAACAATTTGGCAGCA | chr4 | 0.6M | CAPS | TaqI |  |  |
| T-4C0.6ML-1 | ACCTTGGCATAACCAGCATC | chr4 |  | CAPS | TaqI |  |  |
| 4C2.5MU | GTACTCAGTACAGTTTGGTGAG | chr4 | 2.5M | SSR |  |  |  |
| 4C2.5ML | AGAGCATCCCTACTGCTATC | chr4 |  | SSR |  |  |  |
| T-4C5MU-2 | CCATGGTCAAGAAACGGACT | chr4 | 5M | CAPS | TaqI |  |  |
| T-4C5ML-2 | CGGACCACCCAACTAGGTAA | chr4 |  | CAPS | TaqI |  |  |
| 4C7M | ACAAGGGAAAGCAGCAAAAA | chr4 | 7096856 | SSR |  |  |  |
|  | TTGACCTCCTCGTGTCAGTG | chr4 |  | SSR |  |  |  |
| MSAT4.15 | TTTCTTGTCTTTCCCCTGAA | chr4 | W/9362566-9362585 | SSR |  |  |  |
|  | GACGAAGAAGGAGACGAAAA | chr4 | C/9362720-9362739 | SSR |  |  |  |
| T-4C11MU | GCCCGAAACTGGAGTACAAA | chr4 | 11M | CAPS | TaqI |  |  |
| T-4C11ML | TAGACGACGGAGGTTGTGGT | chr4 |  | CAPS | TaqI |  |  |
| T-4C13MU | TCTTTGGTTTGTCTAAAGTTTG | chr4 | 13M | CAPS | TaqI |  |  |
| T-4C13ML | ACAGAGTCGGACAAGGCAAT | chr4 |  | CAPS | TaqI |  |  |
| T-4C15MU | GGCGAATCTGATCTTTGCTC | chr4 | 15M | CAPS | TaqI |  |  |
| T-4C15ML | TCACATTGGCAGGAGACTGA | chr4 |  | CAPS | TaqI |  |  |
| MSAT4.21 | TTATGCTATGGCTGTTTGGT | chr4 | W/17689154-17689173 | SSR |  |  |  |
|  | CGAAATCTGTTCTTGCATTC | chr4 |  | SSR |  |  |  |
| MOJB | TGAAAGATTTTAGGAGGACAA | chr5 | W/2190102-2190122 | SSR |  |  |  |
|  | GTAGGAGAAGGGGACAAGTT | chr5 |  | SSR |  |  |  |
| T-5C5.1MU | GCAACTTGTGGTTTTGTTGTG | chr5 | 5.1M | CAPS | TaqI |  |  |
| T-5C5.1ML | GGCTTCAATTGGGGATATACAA | chr5 |  | CAPS | TaqI |  |  |
| ICE5 | CTTGCAACCGCCAACTCAATCG | chr5 | W/7705245-7705266 | SSR |  |  |  |
|  | CCTGTCTCGCTCCCGCACG | chr5 |  | SSR |  |  |  |
| 5C15M | GGTTCATCCAGTGATGATGGT | chr5 | 15466561 | SSR |  |  |  |
|  | TGAAGTATGCGGAAATCGAA | chr5 |  | SSR |  |  |  |
| CIW9 | CAGACGTATCAAATGACAAATG | chr5 | W/17044001-17044022 | SSR |  |  |  |
|  | GACTACTGCTCAAACTATTCGG | chr5 | C/17044145-17044166 | SSR |  |  |  |
| T-5C24MU | GGTGATCCATGTGTCCCTCT | chr5 | 24M | CAPS | TaqI |  |  |
| T-5C24ML | GGCAGCTGCTATGTTTCCAG | chr5 |  | CAPS | TaqI |  |  |
| T-5C26MU | CGTACATGTGAGGCAATGGA | chr5 | 26M | CAPS | TaqI |  |  |
| T-5C26MUL | AGCAGAGGATGATGCCAGTT | chr5 |  | CAPS | TaqI |  |  |
| T-4C5.1-U | CTGGGAGAGGCTAATCTCCTT | chr4 |  | CAPS | TaqI |  |  |
| T-4C5.1-L | CGGACCACCCAACTAGGTAA | chr4 | C/5097058-5097077 | CAPS | TaqI |  |  |
| T-4C5.5-U | TGGAGAAGCAAATCCAAGAAA | chr4 | W/5474574-5474594 | CAPS | TaqI |  |  |
| T-4C5.5-L | CCTCGTTTACCCTACATGCAC | chr4 | C/5474866-5474886 | CAPS | TaqI |  |  |
| T-4C5.8-U | TGTGCTACTCTCCCTCAGTTG | chr4 | W/5754809-5754832 | CAPS | TaqI |  |  |
| T-4C5.8-L | TTGATGGTGTGAGGGTGAAG | chr4 |  | CAPS | TaqI |  |  |
| T-4C6.12-U | CCTCCATATCGCTCCAAGAT | chr4 | W/6119071-6119090 | CAPS | TaqI |  |  |
| T-4C6.12-L | GCCACCGGATAAATGAAGAA | chr4 | C/6119433-6119452 | CAPS | TaqI |  |  |
| T-4C6.14U | CAAATGATACATCCCCGATTG | chr4 | W/6142589-6142609 | CAPS | HaeIII |  |  |
| T-4C6.14L | TCGTCATTACCGTCAATCCA | chr4 | C/6142920-6142939 | CAPS | TaqI |  |  |
| T-4C6.192U | CCGATCAAGCCTTTTTCAAG | chr4 | W/6192819-6192838 | CAPS | TaqI |  |  |
| T-4C6.192L | CCCTAGTCCGGTAGCATCTG | chr4 | C/6192982-6193001 | CAPS | TaqI |  |  |
| T-4C6.45U | GAGGAAATGCAGGCAAACAT | chr4 |  | CAPS | TaqI |  |  |
| T-4C6.45L | TTGAGCGTTGCGTAAAGATG | chr4 | C/6452856-6452875 | CAPS | TaqI |  |  |
| T-4C6.57-U | TTCGGATTTTATTGTTGTTGTTG | chr4 | W/6572639-6572661 | CAPS | TaqI |  |  |
| T-4C6.57-L | CAAAGCCTTGATGCATCTTAAA | chr4 | C/6572859-6572880 | CAPS | TaqI |  |  |
| T-4C6.74-U | AGAGGCCTCAATTGCTCAAA | chr4 | W/6742063-6742082 | CAPS | TaqI |  |  |
| T-4C6.74-L | CCAACTGATTGCGAGATTCC | chr4 | C/6742435-6742454 | CAPS | TaqI |  |  |
| T-4C6.8-U | CAACTTGGCAGCGTTCTACA | chr4 | W/6857439-6857458 | CAPS | TaqI |  |  |
| T-4C6.8-L | TTCCAGACAGGGATCTCACC | chr4 | C/6857747-6857766 | CAPS | TaqI |  |  |
