# Supplementary material for: AtHKT1 drives adaptation of Arabidopsis thaliana to salinity by reducing floral sodium content
Source: PLoS Genet. 2017 Oct 30;13(10):e1007086. doi: 10.1371/journal.pgen.1007086 (PMC5679648; doi:10.1371/journal.pgen.1007086)
Supplement: S3 Table — (DOCX) [file pgen.1007086.s008.docx]

**S3 Table.** Primers used for sequencing and vector construction.

| **Name** | **Sequence** |
| --- | --- |
| AtHKT1-WTF | AGTAGACCTCTCTACACTTTCC |
| AtHKT1-WTR | TTACTTCCTCAATCCATGGGAG |
| T-DNA LB primer3 | TTGACCATCATACTCATTGCTG |
| HKT1-F4 | ATATCACTTCCTAGATTGTGTC |
| HKT1-R4 | ACTCACATACAAACTGACAATG |
| HKT1-F5 | AAAACATCCACATTGGTGTAC |
| HKT1-R5 | TGATTCTTTCTTGGAATCTCC |
| HKT1-F6 | ACGTGTAATTCACAAAAGTAGC |
| HKT1-R6 | TTACATGGTTAATGAATACCTC |
| HKT1-F7 | AAAAAGAGTGTACTCTGACAC |
| HKT1-R7 | TGGCTTATGTTAGAGACTTTAC |
| HKT1-F8 | TTTGTCAACGTTTGGTGTATC |
| HKT1-R8 | AATTTTCTCGGACAGATTCTC |
| HKT1-F9 | TAGAGTTCTGTAGTGCATACC |
| HKT1-R9 | TAAATTGATGGAAGTGAACGG |
| HKT1-F10 | TAAGACATCCCATGTTACTCC |
| HKT1-R10-2 | TACACGTACATGGAAATTGTC |
| HKT1-F11 | TCTTCCTCTTACAAATACCC |
| HKT1-R11 | AAGAACCACCGAGTACAAGC |
| HKT1-F12 | TGACGTTGAGACTGTTACTG |
| HKT1-R12 | AGAGTACCAAGATAGCTGGG |
| HKT1-F13 | ATACAGCTTCTTTTCTTCTGC |
| HKT1-R13 | TTACTTCCTCAATCCATGGGAG |
| HKT1-F14 | TTAGTGCTATTCTTTTGGCTG |
| HKT1-R14 | TACTCTTAAGTGATGGAGAG |
| HKT1-F15 | AAACATTAACGCCGGAAAAC |
| HKT1-R15 | CTTTCGGTGATTGAAATGAG |
| HKT1-F16 | AGGAGGAGATGATGATTCCG |
| HKT1-R16-3 | TTAATAAATTCTTCCGGTCCC |
| tsu-HKT-R17 | TACGGGTTTAAGTTTTTGGC |
| HKT1-F18 | TTTTATACTACTCCCAACTCG |
| HKT1-R18 | AACGAGGATTAACGATGATGC |
| HKT1-F19-1 | TAATGGCAGTGCATATGGAAAC |
| HKT1-R18 | AACGAGGATTAACGATGATGC |
| HKT1-F19 | TATGGTAGGTTTAAGCAGTTC |
| HKT1-R19 | AAGAAGACATGTGTGTCAAAC |
| UBCF | CTGCGACTCAGGGAATCTTCTAA |
| UBCR | TTGTGCCATTGAATTGAACCC |
| HKT1 exon2-3-1L | CAATCACCGAAAGGCAAAAT |
| HKT1 exon2-3-1R | CGTCCTGCAAACCCATAACT |
| HKT-pro-gene L | GGAGTCGACCAACGAAGCTTTGTTCATAGGTATTTGAGTAAAGAACTCAG |
| PHMS-HKT1-MID-R | GAACATGAGGATAGTGAGGAAGATAAGT |
| PHMS-HKT1-MID-L | TCCTCACTATCCTCATGTTCCTCGGTGG |
| HKT-pro-gene R | CCATTCCTCCTCCTCCTCCGGAAGACGAGGGGTAAAGAATCCATGCGC |
| Tsu-HKT-pro-gene L | GGAGTCGACCAACGAAGCTTCTAAGCTTCATTCTTGTGCAGCCTATTCCTAG |
| PHMS-HKT1-MID-R | GAACATGAGGATAGTGAGGAAGATAAGT |
| PHMS-HKT1-MID-L | TCCTCACTATCCTCATGTTCCTCGGTGG |
| HKT-pro-geneR | CCATTCCTCCTCCTCCTCCGGAAGACGAGGGGTAAAGAATCCATGCGC |
| HKT-GUSL TONG | AAGCTTGCATGCCTGCAGCATTCTTGTGCAGCCTATTCCTAG |
| HKT-GUSR TONG | CTCGGTACCCGGGGATCCTTTAGTTCTCGAGTCGGTCTAAGC |
| HKT-GUSL TONG2 | GCTTGCATGCCTGCAGGCTAATTTGCAATGTTCATAGG |
| HKT-GUSR TONG | CTCGGTACCCGGGGATCCTTTAGTTCTCGAGTCGGTCTAAGC |
| HKTE3-PstI | AACTGCAGTGCATATGGAAACGTGG |
| HKTE3-XbaI | GCTCTAGAGCGGCCAGATTTGGCTG |
| HKTE3-SacI | ACGAGCTCTGCATATGGAAACGTGG |
| HKTE3-NotI | ATAAGAATGCGGCCGCGCGGCCAGATTTGGCTG |
